# New Metabolites and Bioactive Chlorinated Benzophenone Derivatives Produced by a Marine-Derived Fungus *Pestalotiopsis heterocornis*

**DOI:** 10.3390/md15030069

**Published:** 2017-03-13

**Authors:** Hui Lei, Xiuping Lin, Li Han, Jian Ma, Qingjuan Ma, Jialiang Zhong, Yonghong Liu, Tiemin Sun, Jinhui Wang, Xueshi Huang

**Affiliations:** 1 Institute of Microbial Pharmaceuticals, College of Life and Health Sciences, Northeastern University, Shenyang 110819, China; leihui-2008@163.com (H.L.); sherrie525358@126.com (J.M.); maqingjuan0319@163.com (Q.M.); 2 CAS Key Laboratory of Tropical Marine Bio-resources and Ecology, Guangdong Key Laboratory of Marine Materia Medica, RNAM Center for Marine Microbiology, South China Sea Institute of Oceanology, Chinese Academy of Sciences, Guangzhou 510301, China; xiupinglin@hotmail.com (X.L.); yong-hongliu@scsio.ac.cn (Y.L.); 3 Shanghai Institute of Pharmaceutical Industry, Shanghai 201203, China; 13482599366@163.com; 4 Key Laboratory of Structure-Based Drug Design and Discovery, Shenyang Pharmaceutical University, Shenyang 110016, China; suntiemin@126.com (T.S.); 15999290001@163.com (J.W.)

**Keywords:** antibacterial activity, antifungal activity, cytotoxicity, isocoumarin, *Pestalotiopsis heterocornis*

## Abstract

Four new compounds, including two isocoumarins, pestaloisocoumarins A and B (**1**, **2**), one sesquiterpenoid degradation, isopolisin B (**4**), and one furan derivative, pestalotiol A (**5**), together with one known isocoumarin, gamahorin (**3**), and three chlorinated benzophenone derivatives, pestalachloride B (**6**), pestalachloride E (**7**) and a mixture of pestalalactone atropisomers (**8a/8b**), were isolated from a culture of the fungus *Pestalotiopsis heterocornis* associated with sponge *Phakellia fusca*. These new chemical structures were established using NMR and MS spectroscopic data, as well as single-crystal X-ray crystallographic analysis and CD Cotton effects. All of the isolated compounds were evaluated for their antimicrobial and cytotoxic activities. Isocoumarins **1**–**3**, showed antibacterial activities against Gram-positive bacteria *Staphylococcus aureus* and *Bacillus subtilis* with MIC values ranging from 25 to 100 μg/mL and weak antifungal activities. Chlorinated benzophenone derivatives **6**–**8** exhibited antibacterial activities against *S. aureus* and *B. subtilis* with MIC values ranging from 3.0 to 50 μg/mL and cytotoxicities against four human cancer cell lines with IC_50_ values of 6.8–87.8 μM.

## 1. Introduction

Fungi of the genus *Pestalotiopsis* are widely distributed throughout the world and have proven to be a rich source of bioactive natural products [1]. Previous studies on the genus *Pestalotiopsis* have resulted in the isolation of a series of new natural products, including polyketides [2,3,4,5], terpenoids [6,7,8], alkaloids [9,10] and others [11,12], and many of these compounds exhibited various biological activities [2,3,4,5,6,7,8,9,10,11,12].

In recent decades, the genus *Pestalotiopsis* has been isolated as an endophyte from the tropical and subtropical rainforest plants [13]. However, only a few fungi of the genus *Pestalotiopsis* have been reported from marine fauna [14]. Therefore, marine fungi of the genus *Pestalotiopsis* associated with the sponges could be expected to metabolize biologically interesting and chemically diverse compounds.

**Figure 1:**
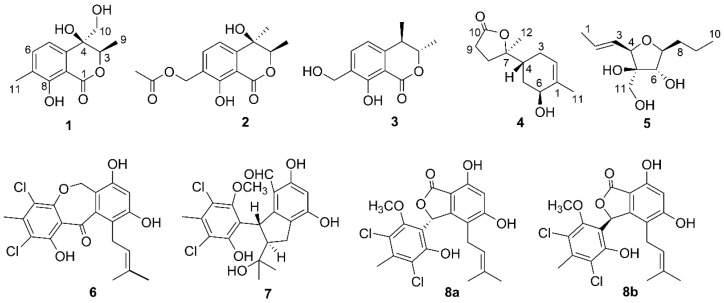
Structures of Compounds **1**–**8**.

Following the above investigations, with the aim of discovering bioactive substances from marine-derived fungi, the secondary metabolites of a culture fermentation of *Pestalotiopsis heterocornis*, which was isolated from the sponge *Phakellia fusca*, were investigated. Four new compounds, including two isocoumarins, pestaloisocoumarin A (**1**) and pestaloisocoumarin B (**2**), one sesquiterpenoid degradation, isopolisin B (**4**), and one furan derivative, pestalotiol A (**5**), together with four known compounds, gamahorin (**3**) [15], pestalachloride B (**6**) [16], pestalachloride E (**7**) [17] and a mixture of pestalalactone atropisomers (**8a/8b**) [18,19], were discovered (Figure 1). The structures of the new compounds were elucidated on the basis of spectroscopic data, circular dichroism (CD) Cotton effects and single-crystal X-ray crystallographic analysis. The cytotoxicities against four human cancer cell lines, antibacterial and antifungal activities against a panel of bacteria and fungi of these isolated compounds were evaluated in the present paper. 

## 2. Results and Discussion

Compound **1** was obtained as a colorless transparent columnar crystal and possessed a molecular formula of C_12_H_14_O_5_ as defined by the ^13^C nuclear magnetic resonance (NMR) and high-resolution electrospray ionization–mass spectrometry (HRESI–MS) data. The ^1^H NMR spectrum (Table 1) displayed signals for two methyls (*δ*_H_ 2.24, s; *δ*_H_ 1.36, d, *J* = 6.6 Hz), an oxygenated methine (*δ*_H_ 4.90, q, *J* = 6.6 Hz) and an oxygenated methylene (*δ*_H_ 3.81, d, *J* = 11.7 Hz; *δ*_H_ 3.55, d, *J* = 11.7 Hz). In addition, two ortho-coupled aromatic protons at *δ*_H_ 7.05 (d, *J* = 7.5 Hz) and *δ*_H_ 7.47 (d, *J* = 7.5 Hz) were observed. The ^13^C NMR spectrum (Table 1) showed 12 carbons, including six aromatic carbons, one oxygenated quaternary carbon, one oxygenated methine, one hydroxymethyl, two methyls and one ester carbonyl carbon. These observations demonstrated that compound **1** was an isocoumarin derivative. Comparing the NMR data of **1** with those of acremonone C revealed their structural similarity [20], except for the different substituent on the benzene ring. Heteronuclear multiple-bond correlation spectroscopy HMBC correlations from H-9 (*δ*_H_ 1.36) to C-3 (*δ*_C_ 78.0) and C-4 (*δ*_C_ 71.3), from H-10 (*δ*_H_ 3.81 and 3.55) to C-3 (*δ*_C_ 78.0), C-4 (*δ*_C_ 71.3) and C-4a (*δ*_C_ 139.2), from H-11 (*δ*_H_ 2.24) to C-6 (*δ*_C_ 136.9), C-7 (*δ*_C_ 125.7) and C-8 (*δ*_C_ 159.5) confirmed the planar structure of **1** as in Figure 1.

**Figure 2:**
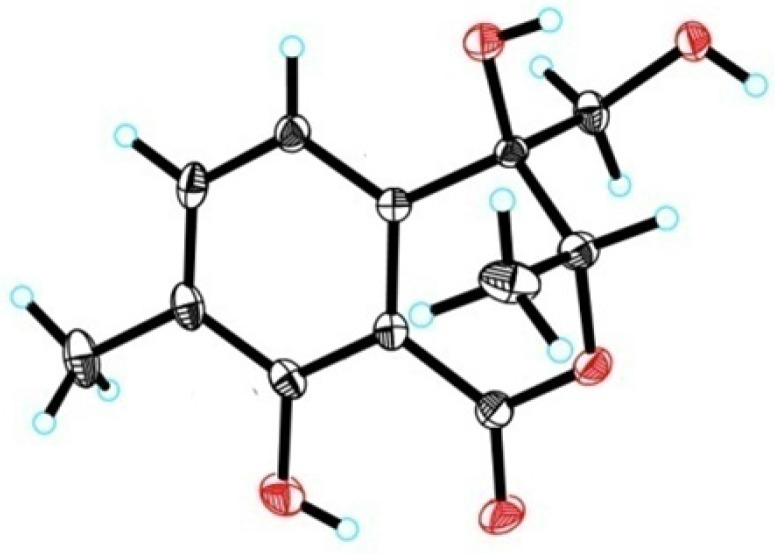
Single crystal X-ray structure of **1**.

**Figure 3:**
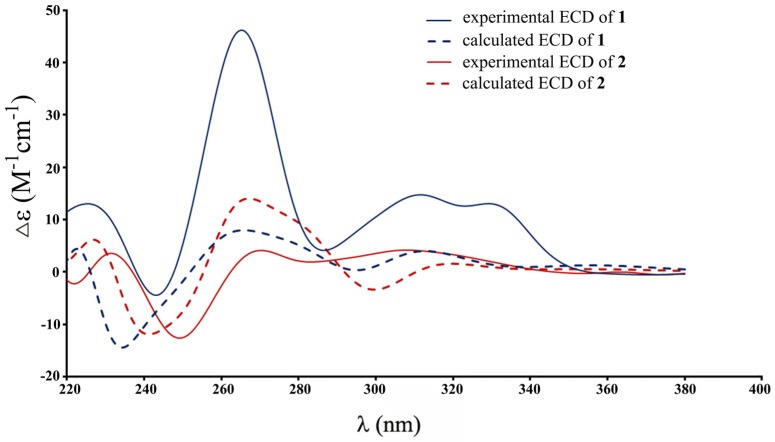
Electronic circular dichroism (ECD) spectra of **1** and **2**.

The relative configuration of **1** was defined on the basis of single-crystal X-ray diffraction analysis (Figure 2). The absolute configuration of **1** was determined by the electronic circular dichroism (ECD) spectra with quantum chemical calculations using the time dependent density functional theory (TDDFT) method at the B3 LYP/6-31 + G(d) level. The calculated ECD spectrum showed the same pattern as the experimental ECD spectrum of **1** (Figure 3). Thus, the absolute configuration of **1** was identified as 3*R* and 4*S* and named pestaloisocoumarin A.

The HRESI–MS analysis of **2** showed a deprotonated ion at *m*/*z* 279.0865 [M − H]^−^. Analysis of the ^1^H and ^13^C NMR data (Table 1) indicated that **2** was very similar to gamahorin (**3**) [15], except that a doublet methyl in **3** was replaced by a singlet methyl in **2**, and an oxygenated quaternary carbon was presented in **2** instead of a methine in **3**. Furthermore, one more acetyl appeared in **2**. HMBC experiments helped to determine the planar structure. HMBC correlations from H-9 (*δ*_H_ 1.43) to C-3 (*δ*_C_ 82.5) and C-4 (*δ*_C_ 67.9), from H-10 (*δ*_H_ 1.57) to C-3 (*δ*_C_ 82.5), C-4 (*δ*_C_ 67.9) and C-4a (*δ*_C_ 145.3), from H-11 (*δ*_H_ 5.17) to C-6 (*δ*_C_ 136.2), C-7 (*δ*_C_ 123.6), C-8 (*δ*_C_ 159.4) and C-13 (*δ*_C_ 171.1) confirmed that the acetyl was linked with C-11 and that C-4 was oxygenated. Nuclear overhauser effect (NOE) correlations between H-3 (*δ*_H_ 4.60) and H-10 (*δ*_H_ 1.57) suggested H-3 and the methyl at C-4 were α-orientated and the methyl at C-3 and the hydroxyl at C-4 were β-orientated. The calculated ECD spectrum of **2** showed excellent agreement with experimental results, and the 3*R* and 4*R* configurations of **2** was confirmed (Figure 3). Thus, Compound **2** was determined and named pestaloisocoumarin B.

**Figure 4:**
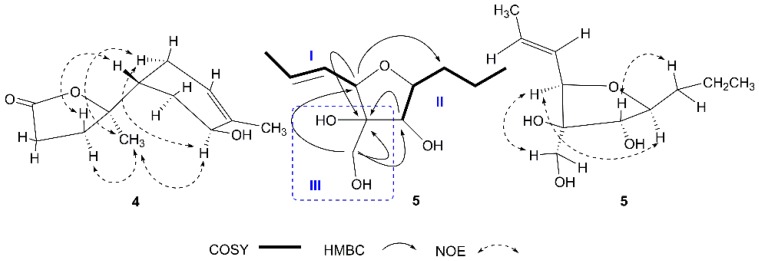
^1^H-^1^H COSY (correlation spectroscopy) HMBC (heteronuclear multiple-bond correlation spectroscopy) correlations of **5** and NOE (nuclear overhauser effect) correlations of **4** and **5**. Fragment I: -CH-CH=CH-CH_3_; Fragment II: -CH(OH)CHCH_2_CH_2_CH_3_; Fragment III: The moiety in the blue dotted line box.

Compound **4** was isolated as a colorless oil and gave the molecular formula C_12_H_18_O_3_ from HRESI–MS data. The ^1^H NMR and ^13^C NMR data (Table 2) of **4** were almost identical to those of the known compound polisin B [21], which is a 11,12,15-norbisabolane sesquiterpenoid. Analysis of the correlation spectroscopy (COSY), heteronuclear single quantum coherence (HSQC) and HMBC correlations exhibited that **4** was a isomer of polisin B. Except for carbons at C-1 (Δ*δ*_C_ +2.0), C-2 (Δ*δ*_C_ −1.2), C-4 (Δ*δ*_C_ +5.2), C-5 (Δ*δ*_C_ +1.3), C-6 (Δ*δ*_C_ +2.4) and C-11 (Δ*δ*_C_ −2.2), the carbons in the lactone ring of **4** presented the same ^13^C NMR data with those of polisin B, which indicated that **4** possesses the same relative configuration at C-4 and C-7 as polisin B. NOE correlations observed from H-12 (*δ*_H_ 1.37) to H-8α (*δ*_H_ 1.98), H-3α (*δ*_H_ 1.81) and H-6 (*δ*_H_ 4.00), from H-6 (*δ*_H_ 4.00) to H-3α (*δ*_H_ 1.81), from H-4 (*δ*_H_ 2.06) to H-8*β* (*δ*_H_ 2.24) indicated that H-4 and 6-OH were β-orientated and that H-6 and the methyl at C-7 were α-orientated (Figure 4). Thus, the structure of **4** was assigned and named isopolisin B.

Compound **5** was obtained as colorless oil with the molecular formula C_11_H_20_O_4_ as determined by HRESI–MS. The ^1^H NMR spectrum (Table 2) showed five methines, including two olefinic methines at *δ*_H_ 5.78, 5.60, three oxygenated methines at *δ*_H_ 4.13, 3.75, 3.58, three methylenes, including one oxygenated methylene at *δ*_H_ 3.69, 3.51 and two methyls at *δ*_H_ 1.75, 0.96. COSY correlations between H-1/H-2/H-3/H-4 and between H-6/H-7/H-8/H-9/H-10 gave two fragments I and II. HMBC correlations between H-11 (*δ*_H_ 3.69, 3.51) and C-5 (*δ*_C_ 81.6) deduced a Fragment III as in Figure 4. HMBC correlations between H-4 (*δ*_H_ 4.13) and C-5 (*δ*_C_ 81.6), C-8 (*δ*_C_ 35.9), between H-6 (*δ*_H_ 3.75) and C-5 (*δ*_C_ 81.6), between H-11 (*δ*_H_ 3.69, 3.51) and C-4 (*δ*_C_ 82.9), C-5 (*δ*_C_ 81.6), C-6 (*δ*_C_ 82.7) confirmed the linkage between Fragments I, II and III (Figure 4). NOE correlations from H-4 (*δ*_H_ 4.13) to H-7 (*δ*_H_ 3.58), H-11 (*δ*_H_ 3.69, 3.51), from H-6 (*δ*_H_ 3.75) to H-8 (*δ*_H_ 1.65) indicated that H-4, H-7 and the hydroxymethyl at C-5 were cis and H-6 and the propyl at C-7 were cis configurations (Figure 4). The configuration of the double bond of **5** was confirmed as *E* geometry based on the coupling constant values between H-2 and H-3 (*J*_H2,3_ = 14.7 Hz). Therefore, the structure of **5** was elucidated and named pestalotiol A.

Compounds **3**, **6**–**8** were identified as gamahorin (**3**) [15], pestalachloride B (**6**) [16], pestalachloride E (**7**) [17] and a mixture of pestalalactone atropisomers (**8a**/**8b**) [18,19] by comparison of the ^1^H NMR, ^13^C NMR and mass spectroscopy MS data with those reported.

All of the isolated compounds were evaluated for their cytotoxic activities against four human cancer cell lines via the 3-(4,5-dimethylthiazol-2-yl)-2,5-diphenyltetrazolium bromide assay (MTT) assay (Table 3) and antimicrobial activities against three bacteria and three fungi using a micro broth dilution method (Table 4). Chlorinated benzophenone derivatives **6**, **7** and a mixture of **8a**/**8b** exhibited moderate cytotoxicities against four human cancer cell lines with half maximal inhibitory concentration (IC_50_) values 6.8–87.8 μM; while Compounds **1**–**5** did not show an obvious inhibition effect against any test cancer cell lines at 100 μM. Isocoumarins **1**–**3** and chlorinated benzophenone derivatives **6**–**8** showed antibacterial activities against Gram-positive bacteria *Staphylococcus aureus* and *Bacillus subtilis* with minimum inhibitory concentration (MIC) values ranging from 3 to 100 μg/mL. Among them, isocoumarins **1**–**3** also exhibited weak antifungal activities against three test fungi or part of them with MIC values 100 μg/mL. Compounds **6**–**8** were inactive against three test fungi at 100 μg/mL, and Compounds **4**, **5** did not show antimicrobial activity against any test microorganism at 100 μg/mL.

## 3. Experimental Section

### 3.1. General Experimental Procedures

Optical rotations were determined using an AntonPaar MCP200 automatic polarimeter (Anton Paar Ltd., Graz, Austria). Ultraviolet spectra were measured with a BeckmanCoulter DU 730 nucleic acid/protein analyzer (Beckman Coulter, Inc., Brea, CA, USA). Infra-red (IR) spectra were recorded with a Bruker Tensor 27 FTIR spectrometer (film) (Bruker Optics, Ettlingen, Germany). 1D and 2D NMR spectra were collected on a Bruker AV-600 spectrometer (Bruker, Rheinstetten, Germany), *δ* in ppm rel. to tetramethylsilane (TMS), *J* in Hz. ESIMS were recorded on an Agilent 1290-6420 Triple Quadrupole LC–MS spectrometer (Agilent Technologies, Santa Clara, CA, USA). HRESI–MS were performed using a BrukerMicroTOF-Q mass spectrometer (Bruker, Daltonics, Billerica, MA, USA). Silica gel (100–200 mesh, 200–300 mesh, Qingdao Marine Chemical Ltd., Qingdao, China), Sephadex LH-20 (GE Healthcare Bio-sciences AB, Uppsala, Sweden) and YMC*GEL ODS-A (S-50 μm, 12 nm) (YMC Co., Ltd., Kyoto, Japan) were used for column chromatography. Semipreparative high performance liquid chromatography (HPLC) was performed using an Octadecylsilyl silica (ODS) column (250 × 10 mm, 5 μm, YMC-ODS-A). CD spectra were measured on a Biologic MOS-450 spectra polarimeter (Biologic Science, Claix, France). X-ray crystallographic analysis was carried out on a Bruker SMART APEX-II diffractometer (Bruker Biospin Group, Karlstuhe, Germany). MTT and antimicrobial assays were analyzed using a microplate reader (BioTek Synergy H1, BioTek Instruments, Inc., Vermont, VT, USA).

### 3.2. Fungal Material

The fungal strain, *P. heterocornis*, was isolated from the sponge *P. fusca*, which was collected from the Xisha Islands of China in 2012. The strain was identified by Xiuping Lin, and a voucher specimen (No. XWS03F09) was deposited in the CAS Key Laboratory of Tropical Marine Bio-resources and Ecology, South China Sea Institute of Oceanology, Chinese Academy of Sciences, Guangzhou, China.

### 3.3. Fermentation, Extraction and Isolation

The fungal strain *P. heterocornis* was cultivated in 1000 mL conical flasks containing solid rice medium (each flask contained 200 g of rice, 5 g of artificial sea salt; 200 mL of distilled water, boiled in an autoclave for 20 min at 121 °C, at 28 °C without shaking for 36 days. The total of rice culture was extracted with EtOAc three times. The combined EtOAc extract was evaporated to dryness under reduced pressure to afford 182.5 g of crude extract.

The extract was subjected to silica gel column chromatography (CC) (CH_2_Cl_2_/MeOH *v*/*v*, 50:1–0:100) to yield 6 fractions (Frs. 1–6). Fraction 3 was subjected to Sephadex LH-20 chromatography (MeOH) to produce three subfractions (Frs. 3.1–3.3). Fr. 3.1 was isolated by CC on silica gel eluted with CH_2_Cl_2_/acetone (15:1 to 0:1, *v*/*v*) to afford five subfractions (Frs. 3.1.1–3.1.5). Fr. 3.1.2 was further separated by ODS CC, eluting with MeOH/water (85%) and then purified by preparative TLC (CH_2_Cl_2_/MeOH, 20:1) to yield Compound **4** (8.5 mg). Fr. 3.1.4 was further separated by ODS CC, eluting with MeOH/water (70%) to give Compound **5** (7.5 mg). Fraction 4 was separated using silica gel column chromatography eluting with CH_2_Cl_2_/acetone (10:1) to yield five subfractions (Frs. 4.1–4.5). Fr. 4.5 was subjected to repeated column chromatography (Sephadex LH-20 and ODS) and further purified by semipreparative HPLC (65% MeOH/H_2_O) to give Compounds **6** (7.0 mg), **7** (5.0 mg) and a mixture of **8a**/**8b** (2.0 mg), respectively. Fraction 6 was fractionated on Sephadex LH-20 eluted with MeOH to produce 3 fractions (Frs. 6.1–6.3). Fr. 6.2 was separated by silica gel column chromatography, eluting with CH_2_Cl_2_/acetone (4:1) to yield four subfractions (Frs. 6.2.1–6.2.4). Fr. 6.2.3 was further purified by HPLC (45% MeOH/H_2_O) to give Compounds **1** (4.5 mg), **2** (8.0 mg) and **3** (8.0 mg), respectively.

Pestaloisocoumarin A (**1**): colorless transparent columnar crystal; ${\left[\alpha \right]}_{D}^{20}$ +13.3 (*c* 0.60, MeOH); UV (MeOH) λ_max_ (log ε) 249 (4.51), 321 (4.37) nm; IR (film) ν_max_ 3301, 2922, 2852, 1727, 1667, 1617, 1459, 1421, 1247, 1123, 1063 cm^−1^; CD λ_max_ (∆ε) 243 (−4.45), 265 (46.16), 312 (17.72); ^1^H NMR and ^13^C NMR data, see Table 1; HRESI–MS *m*/*z* 237.0786 [M − H]^−^ (calcd. for C_12_H_13_O_5_, 237.0763).

Pestaloisocoumarin B (**2**): white amorphous solid; ${\left[\alpha \right]}_{D}^{20}$ −16.0 (*c* 0.50, MeOH); UV (MeOH) λ_max_ (log ε) 246 (4.32), 321 (4.27) nm; IR (film) ν_max_ 3404, 2991, 2920, 2852, 1736, 1669, 1623, 1431, 1382, 1246, 1158, 1048 cm^−1^; CD λ_max_ (∆ε) 249 (−12.63), 270 (4.11), 308 (4.17); ^1^H NMR and ^13^CNMR data, see Table 1; HRESI–MS *m*/*z* 279.0865 [M − H]^−^ (calcd. for C_14_H_15_O_6_, 279.0869).

Isopolisin B (**4**): colorless oil; ${\left[\alpha \right]}_{D}^{20}$ −19.0 (*c* 0.84, MeOH); IR (film) ν_max_ 3414.6, 2922.3, 2855.4, 1763.0, 1672.4, 1605.4, 1543.5, 1451.9, 1383.0, 1288.0, 1259.1, 1203.6, 1148.3, 1035.2, 965.5 cm^−1^; ^1^H NMR and ^13^C NMR data, see Table 2; HRESI–MS *m*/*z* 233.1292 [M + Na]^+^ (calcd. for C_12_H_18_O_3_Na, 233.1154).

Pestalotiol A (**5**): colorless oil; ${\left[\alpha \right]}_{D}^{20}$ +28.6 (*c* 0.70, MeOH); IR (film) ν_max_ 3379.5, 2958.2, 2928.2, 2872.4, 1667.0, 1604.8, 1452.8, 1408.4, 1378.4, 1089.8, 1041.6, 969.1, 851.7 cm^−1^; ^1^H NMR and ^13^C NMR data, see Table 2; HRESI–MS *m*/*z* 239.1394 [M + Na]^+^ (calcd. for C_11_H_20_O_4_Na, 239.1259).

### 3.4. X-ray Crystallography of ***1***

Colorless transparent columnar crystals of **1** were obtained from a 10:1 (*v*/*v*) mixture of MeOH and H_2_O. Crystal data of **1**, C_12_H_14_O_5_, MW = 238.23, monoclinic, crystal size 0.10 × 0.10 × 0.08 mm, space group P21, unit cell dimensions a = 7.5403 (2) Å, b = 12.2371 (4) Å, c = 12.5115 (4) Å, α = 90°, β = 99.153 (2)°, γ = 90°, volume = 1139.75 (6) Å^3^, Z = 4 ρ calcd. = 1.388 g/cm^3^, θ range = 3.58–68.28°, Mo Kα radiation, wavelength = 1.54178 Å, temperature = 173 K, F (000) = 504, reflections collected 11,422, unique 4032 [R(int) = 0.0325], completeness to θ = 68.28°, 99.7%, the final refinement gave R1 = 0.0455 and wR2 = 0.1250 (w = 1/σ|F|^2^), S = 1.054, maximum transmission 0.7531, minimum transmission 0.7323, absolute structure parameter = 0.1 (2).

Bruker SMART APEX-II data collection, the structures were solved by direct methods using SHELXS-97 and refined by means of full-matrix least squares on F^2^.

### 3.5. Computational Work

The stable conformational analysis was carried out with SYBYL software (Tripos, San Francisco, CA, USA) using the MMFF94S (Merck Molecular Force Field 94S) force field with an energy cutoff of 10 kcal/mol. The stable conformers were used for geometry optimization at the B3 LYP/6-31G(d) level with a CPCM (conductor-like conductor polarizable continuum model) solvent model for methanol in the Gaussian 09 software. The ECD spectra of stable conformers were then calculated based on the TDDFT method at the B3 LYP/6-31 + G(d) level in methanol [22]. The final ECD curves were generated based on the rotatory strengths with a half-band of 0.3 eV by SpecDis [23] and calculated from the spectra of individual conformers according to their contribution to the Boltzmann weighting. The theoretical spectra have been corrected based on the UV correction. 

### 3.6. Cytotoxicity Assay

The cytotoxicities of **1**–**8** were evaluated against four human carcinoma cell lines, including a human gastric carcinoma cell line (BGC-823), a human large-cell lung carcinoma cell line (H460), a human prostate cancer cell line (PC-3) and a human hepatocellular carcinoma cell line (SMMC-7721) in an MTT assay as previously reported [24]. The IC_50_ value was defined as a 50% reduction of absorbance from the control assay. Adriamycin (Sigma Inc., St Louis, MO, USA) was assayed as a positive control.

### 3.7. Antimicrobial Assay

A micro broth dilution assay as previously reported [25] was used to evaluate the MICs of **1**–**8** against three bacteria (*Bacillus subtilis* ATCC 6633, *Staphylococcus aureus* ATCC 25923 and *Escherichia coli* ATCC 25922) and three fungi (*Candida albicans* MYA-2867, *Candida parapsilosis* ATCC 22019 and *Cryptococcus neoformans* ATCC 208821). The MIC was defined as the lowest concentration of the antimicrobial agent that completely inhibited visual growth of an organism. Ciprofloxacin and amphotericin B (Sigma Inc.) were used as positive controls against bacteria and fungi, respectively.

## 4. Conclusions

In this study, eight compounds, including four new metabolites, were isolated from the marine-derived fungus *P. heterocornis*. The structures of the isolated compounds were elucidated by the detailed analysis of spectroscopic data, as well as single-crystal X-ray crystallographic analysis and CD Cotton effects. All compounds were evaluated for their antibacterial and cytotoxic activities. Isocoumarins **1**–**3** showed antimicrobial activities against both Gram-positive bacteria and fungi. Chlorinated benzophenone derivatives **6**–**8** showed cytotoxicities and antibacterial activities against Gram positive bacteria.

## Figures and Tables

**Table 1:** ^1^H nuclear magnetic resonance (NMR) (600 MHz) and ^13^C NMR (150 MHz) data for Compounds **1** and **2** in CD_3_OD.

	1		2	
Position	*δ*_C_, type	*δ*_H_ (*J* in Hz)	*δ*_C_, type	*δ*_H_ (*J* in Hz)
1	168.9, C		169.0, C	
3	78.0, CH	4.90, q (6.6)	82.5, CH	4.60, q (6.6)
4	71.3, C		67.9, C	
4a	139.2, C		145.3, C	
5	115.3, CH	7.05, d (7.5)	114.2, CH	7.16, d (7.7)
6	136.9, CH	7.47, d (7.5)	136.2, CH	7.64, d (7.7)
7	125.7, C		123.6, C	
8	159.5, C		159.4, C	
8a	106.1, C		106.4, C	
9	13.6, CH_3_	1.36, d (6.6)	13.5, CH_3_	1.43, d (6.6)
10	65.7, CH_2_	3.81, d (11.7) 3.55, d (11.7)	23.6, CH_3_	1.57, s
11	14.0, CH_3_	2.24, s	60.3, CH_2_	5.17, s
AcO			171.1, C	
			19.2, CH_3_	2.08, s

**Table 2:** ^1^H NMR (600 MHz) and ^13^C NMR (150 MHz) data for Compounds **4** and **5**.

	4 (in CD_3_OD)		4 (in CDCl_3_)		5 (in CD_3_OD)	
Position	*δ*_C_, type	*δ*_H_ (*J* in Hz)	*δ*_C_, type	*δ*_H_ (*J* in Hz)	*δ*_C_, type	*δ*_H_ (*J* in Hz)
1	134.4, C		134.7, C		16.7, CH_3_	1.75, dd (6.4, 1.5)
2	123.5, CH	5.57, brd (5.0)	124.2, CH	5.57, brd (4.4)	130.4, CH	5.78, dd (14.7, 6.6)
3	26.1, CH_2_	2.10, m 1.81, m	26.5, CH_2_	2.12, m 1.82, m	126.3, CH	5.60, brdd (14.7, 7.2)
4	37.2, CH	2.06, m	37.4, CH	2.03, m	82.9, CH	4.13, d (7.9)
5	32.3, CH_2_	1.95, brd (13.0) 1.48, td (13.0, 4.0)	32.5, CH_2_	1.97, brd (13.2) 1.49, td (13.2, 3.7)	81.6, C	
6	67.1, CH	4.00, brs	68.0, CH	4.07, brs	82.7, CH	3.75, d (4.5)
7	89.0, C		88.3, C		84.7, CH	3.58, td (6.6, 4.5)
8	30.5, CH_2_	2.24, ddd (13.0, 10.0, 8.8) 1.98, ddd (13.0, 10.2, 4.7)	31.2, CH_2_	2.19, ddd (13.2, 10.1, 9.0) 1.94, ddd (13.2, 10.0, 4.6)	35.9, CH_2_	1.65, m
9	28.4, CH_2_	2.72, ddd (18.3, 10.2, 8.8) 2.58, ddd (18.3, 10.0, 4.7)	29.0, CH_2_	2.65, ddd (18.2, 10.0, 9.0) 2.57, ddd (18.2, 10.1, 4.6)	18.9, CH_2_	1.50, m 1.42, m
10	178.1, C		176.7, C		13.0, CH_3_	0.96, t (7.4)
11	19.6, CH_3_	1.78, s	20.8, CH_3_	1.80, s	62.6, CH_2_	3.69, d (11.3) 3.51, d (11.3)
12	21.5, CH_3_	1.37, s	23.1, CH_3_	1.37, s		
OH				3.65, brs		

**Table 3:** Cytotoxic activities of Compounds **6**–**8** (half maximal inhibitory concentration, IC_50_ in μM).

Compound	BGC-823	H460	PC-3	SMMC-7721
**6**	6.8	23.6	28.1	7.9
**7**	48.0	87.8	55.1	40.2
**8a/8b**	53.8	48.2	66.1	41.5
Adriamycin	1.5	1.0	1.8	2.2

**Table 4:** Antimicrobial activities of Compounds **1**–**3**, **6**–**8** (minimum inhibitory concentration, MIC μg/mL).

MIC	1	2	3	6	7	8a/8b	Control
*Bacillus subtilis*	50	25	100	3	50	50	0.25 *^a^*
*Staphylococcus aureus*	25	25	100	3	25	50	0.13 *^a^*
*Escherichia coli*	-	-	-	-	-	-	0.13 *^a^*
*Candida albicans*	100	-	-	-	-	-	1.0 *^b^*
*Candidad parapsilosis*	100	-	100	-	-	-	2.0 *^b^*
*Cryptococcus neoformans*	100	100	100	-	-	-	2.0 *^b^*

*^a^* Ciprofloxacin; *^b^* Amphotericin B.

## Supplementary Materials

The materials (1D and 2D NMR, HRESI–MS spectra of Compounds **1**–**2**, **4**, **5**) are available at www.mdpi.com/1660-3397/15/3/69/s1.
