# Supplementary material for: New Metabolites and Bioactive Chlorinated Benzophenone Derivatives Produced by a Marine-Derived Fungus Pestalotiopsis heterocornis
Source: Mar Drugs. 2017 Mar 13;15(3):69. doi: 10.3390/md15030069 (PMC5367026; doi:10.3390/md15030069)
Supplement: Supplementary file 1 [file marinedrugs-15-00069-s001.pdf]

Mar. Drugs **2017**, 15, 69

New Metabolites and Bioactive Chlorinated Benzophenone Derivatives Produced by a Marine-Derived Fungus *Pestalotiopsis heterocornis*

Hui Lei [1], Xiuping Lin [2], Li Han [1,*], Jian Ma [1], Qingjuan Ma [1], Jialiang Zhong [3], Yonghong Liu [2] Tiemin Sun [4], Jinhui Wang [4] and Xueshi Huang [1,*]

Mass Spectrum Molecular Formula Report

Analysis Info

Analysis Name	D:\Data\20160322ceyang\neu-27_1-A,5_01_6802.d	Acquisition Date	3/22/2016 4:30:47 PM
Method	20131026_ceyang.m	Operator	Bruker Customer
Sample Name	neu-27	Instrument / Ser#	micrOTOF-Q 125
Comment			

Acquisition Parameter

Source Type	ESI	Ion Polarity	Negative	Set Nebulizer	1.2 Bar
Focus	Not active	Set Capillary	3800 V	Set Dry Heater	180 °C
Scan Begin	50 m/z	Set End Plate Offset	-500 V	Set Dry Gas	8.0 l/min
Scan End	1500 m/z	Set Collision Cell RF	400.0 Vpp	Set Divert Valve	Source

Generate Molecular Formula Parameter

Formula, min.	C12H13O5				
Formula, max.					
Measured m/z	237.079	Tolerance	8 ppm	Charge	-1
Check Valence	no	Minimum	0	Maximum	0
Nirogen Rule	no	Electron Configuration	both		
Filter H/C Ratio	no	Minimum	0	Maximum	3
Estimate Carbon	yes				

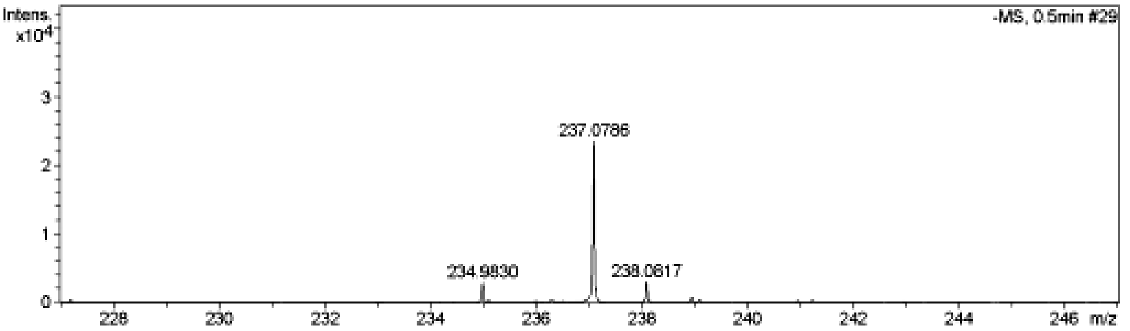

Sum Formula	Sigma	m/z	Err [ppm]	Mean Err [ppm]	Err [mDa]	rdb	N Rule	e⁻
C 12 H 13 O 5	0.003	237.0768	-7.96	-7.72	-1.89	6.50	ok	even

Figure S1. High-resolution electrospray ionization–mass spectrometry (HRESI–MS) spectrum of new compound **1**

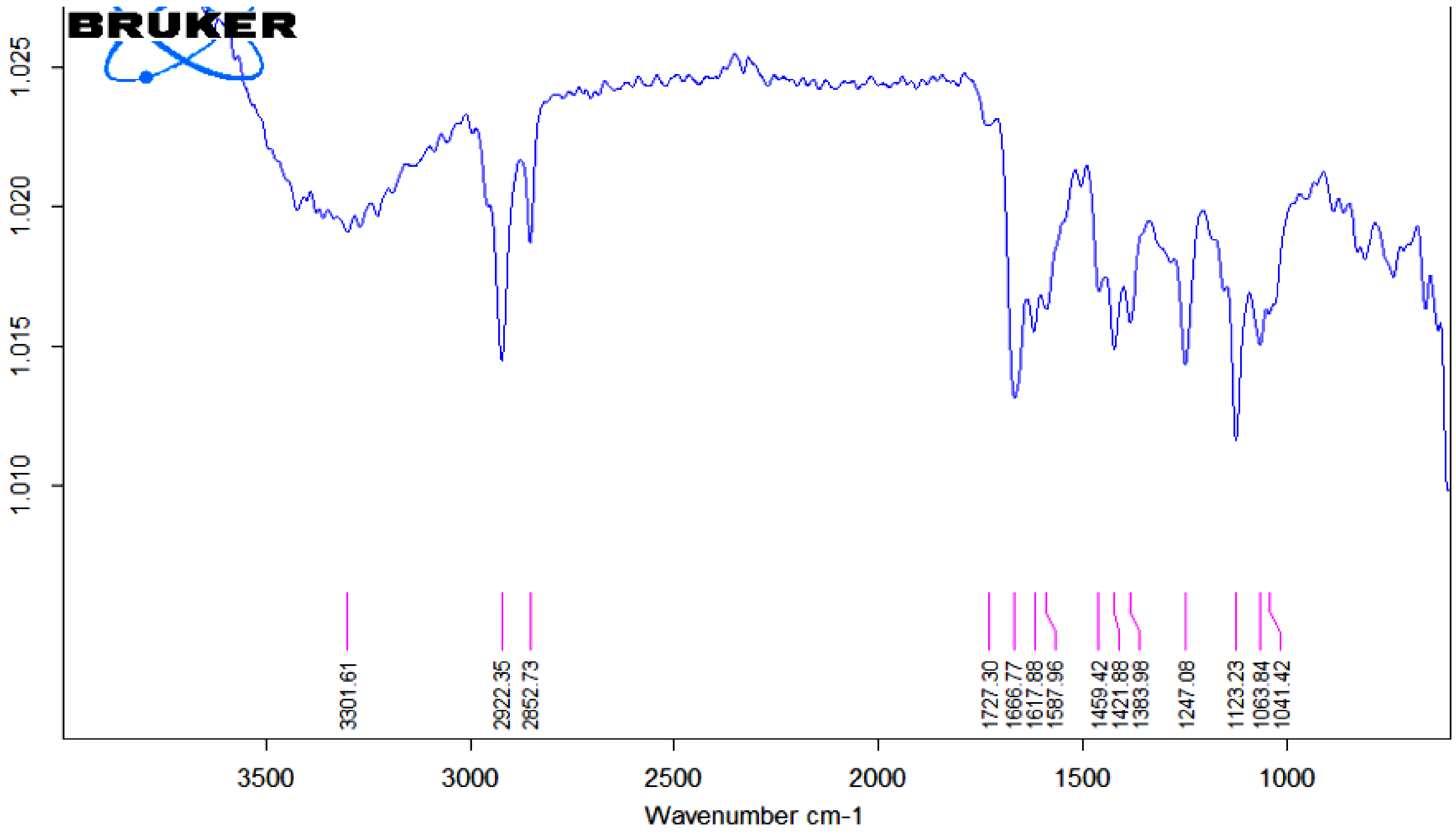

Figure S2. Infra-red (IR) spectrum of new compound **1**

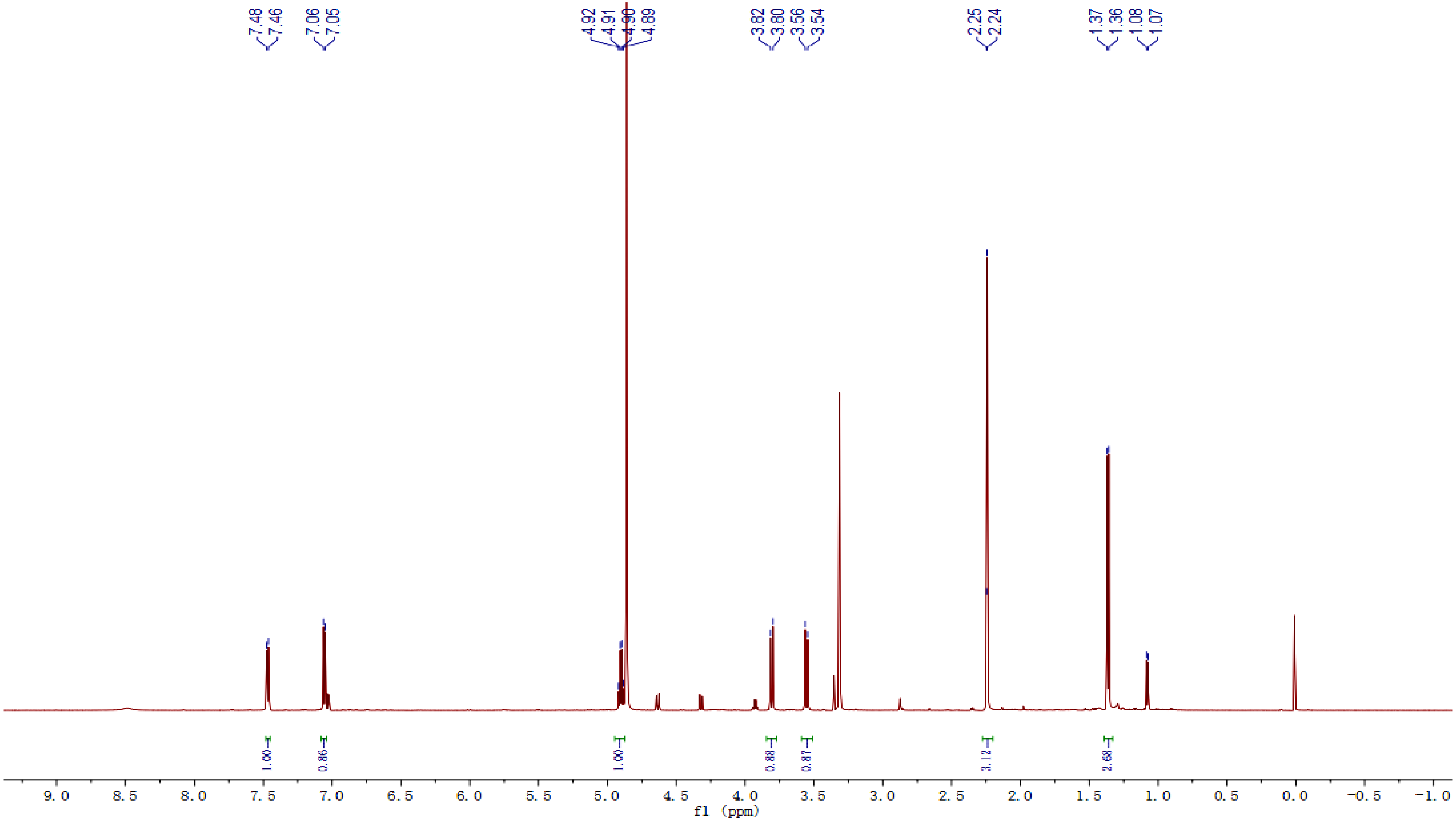

Figure S3. ^{1}H nuclear magnetic resonance (NMR) (600 MHz, CD_3OD) spectrum of new compound **1**

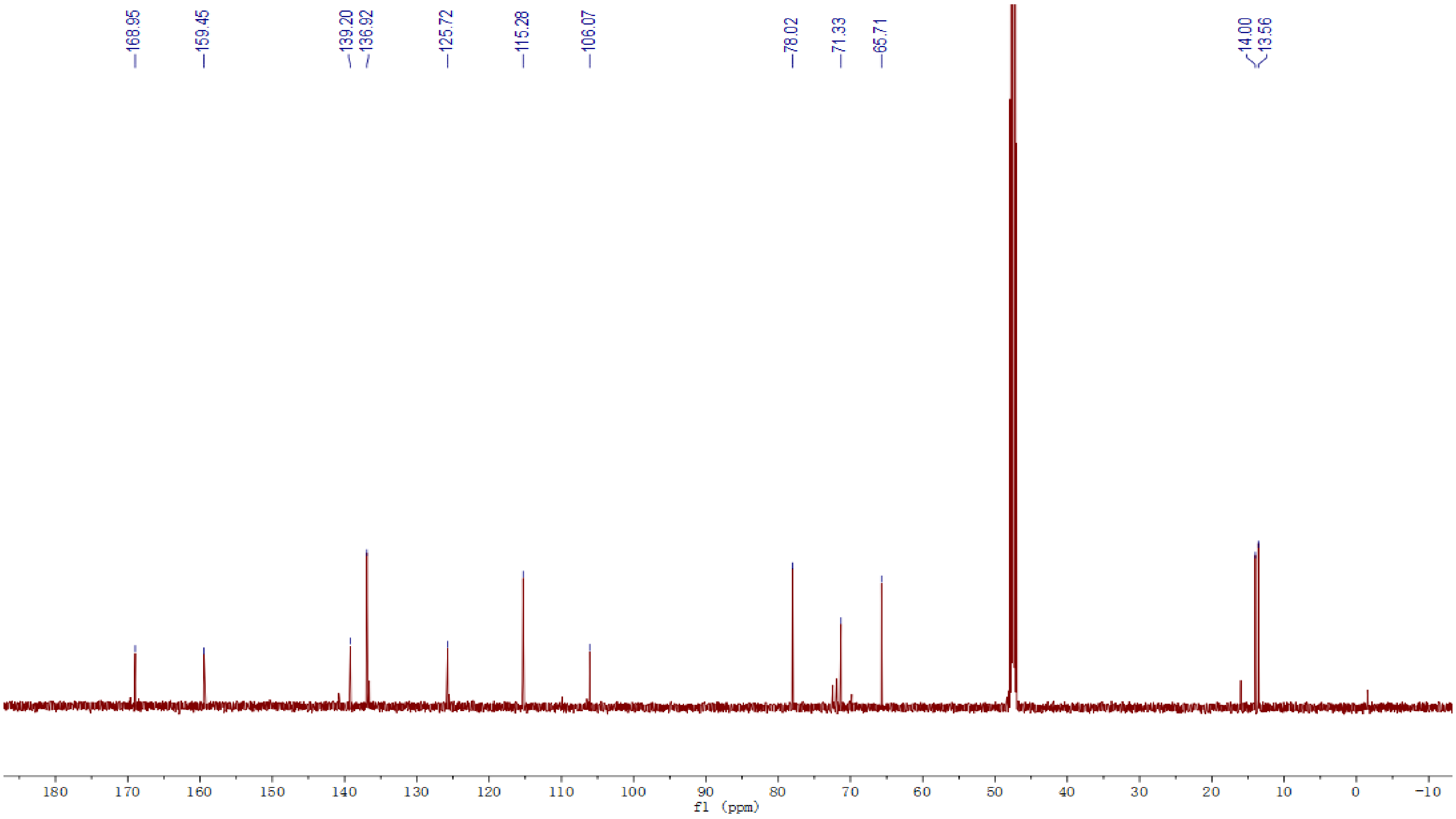

Figure S4. ^{13}C NMR (150 MHz, CD_3OD) spectrum of new compound **1**

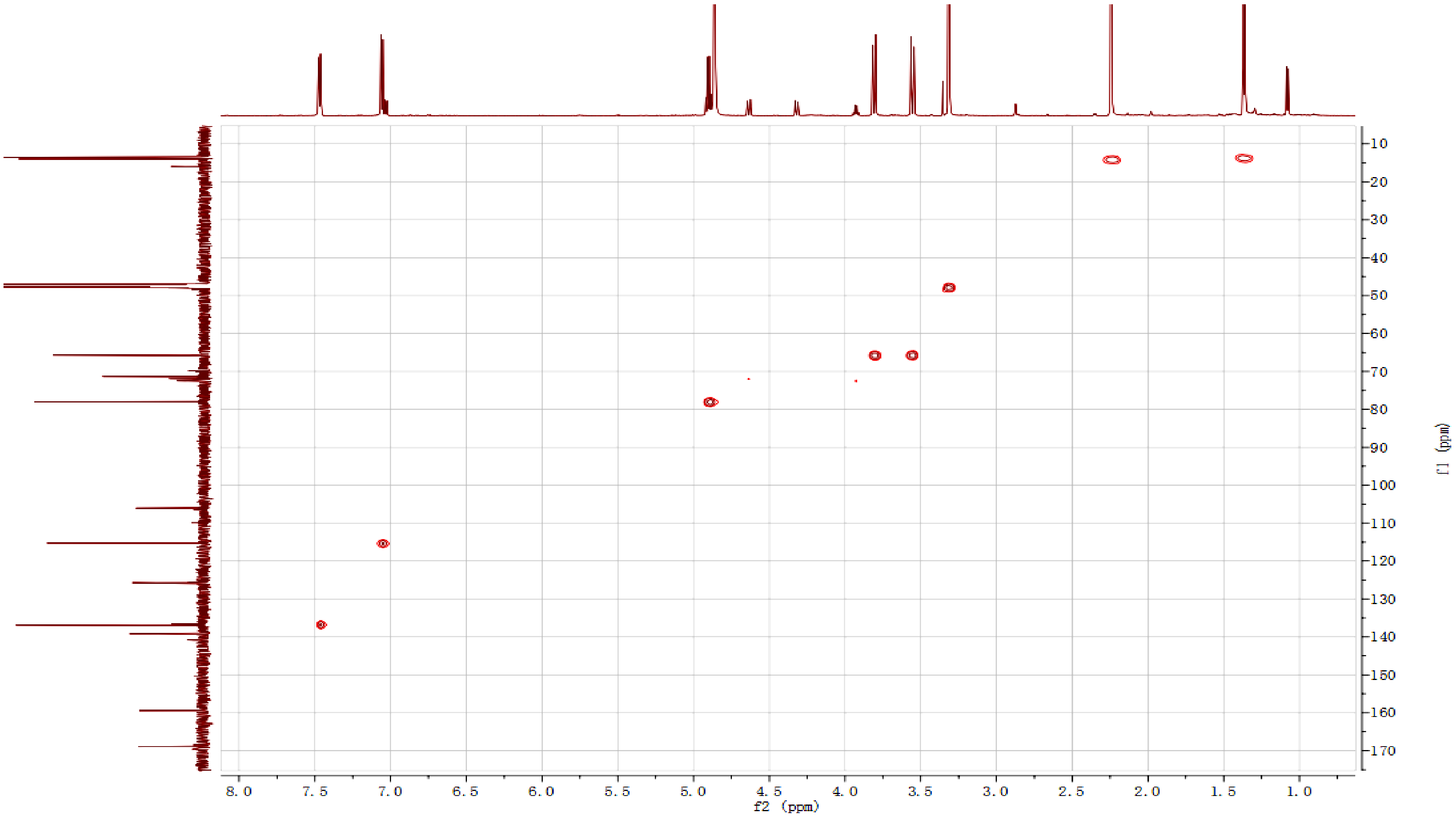

Figure S5. Heteronuclear single quantum coherence (HSQC) spectrum of new compound **1**

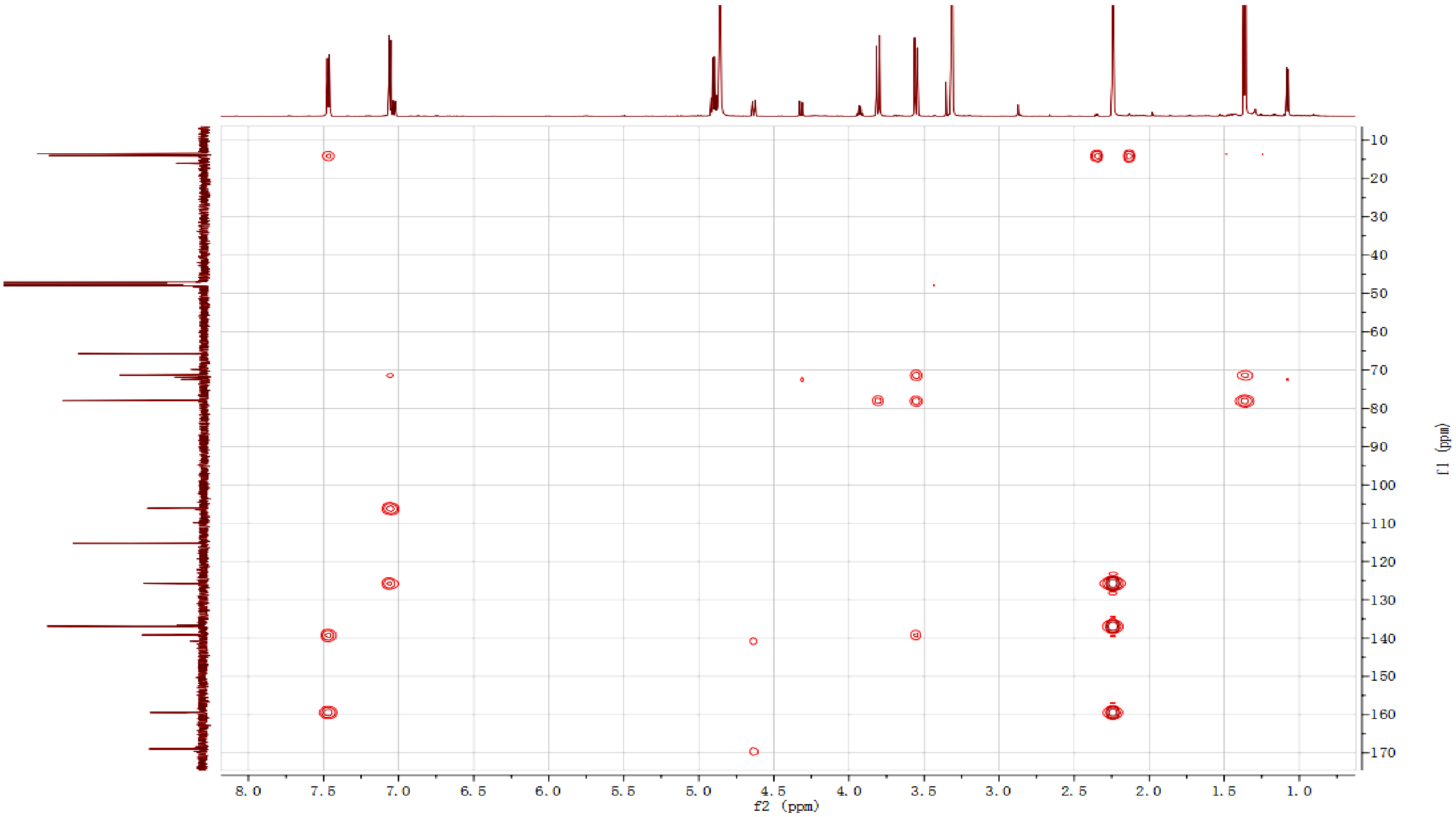

Figure S6. Heteronuclear multiple-bond correlation spectroscopy (HMBC) spectrum of new compound **1**

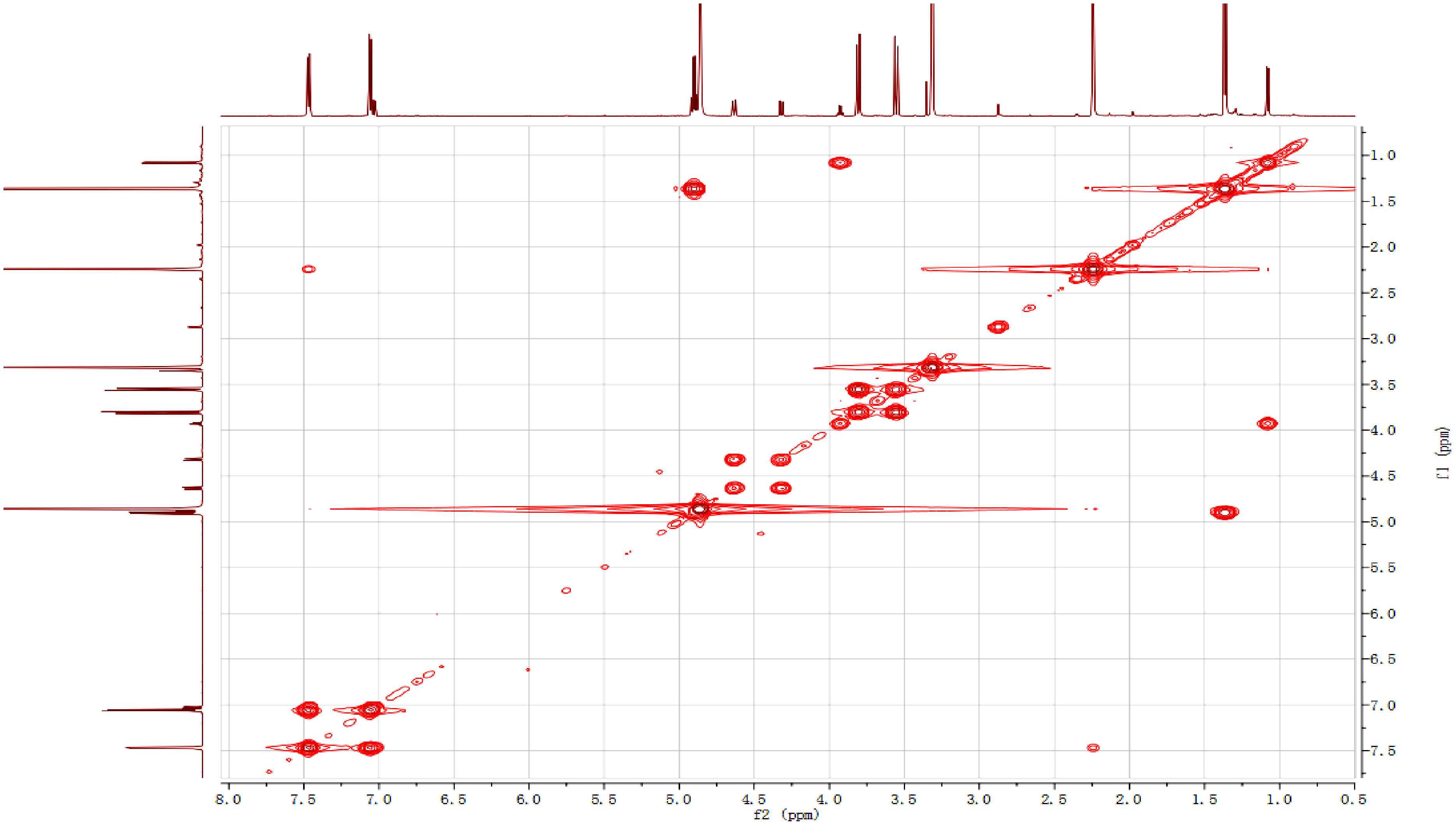

Figure S7. Correlation spectroscopy (COSY) spectrum of new compound **1**

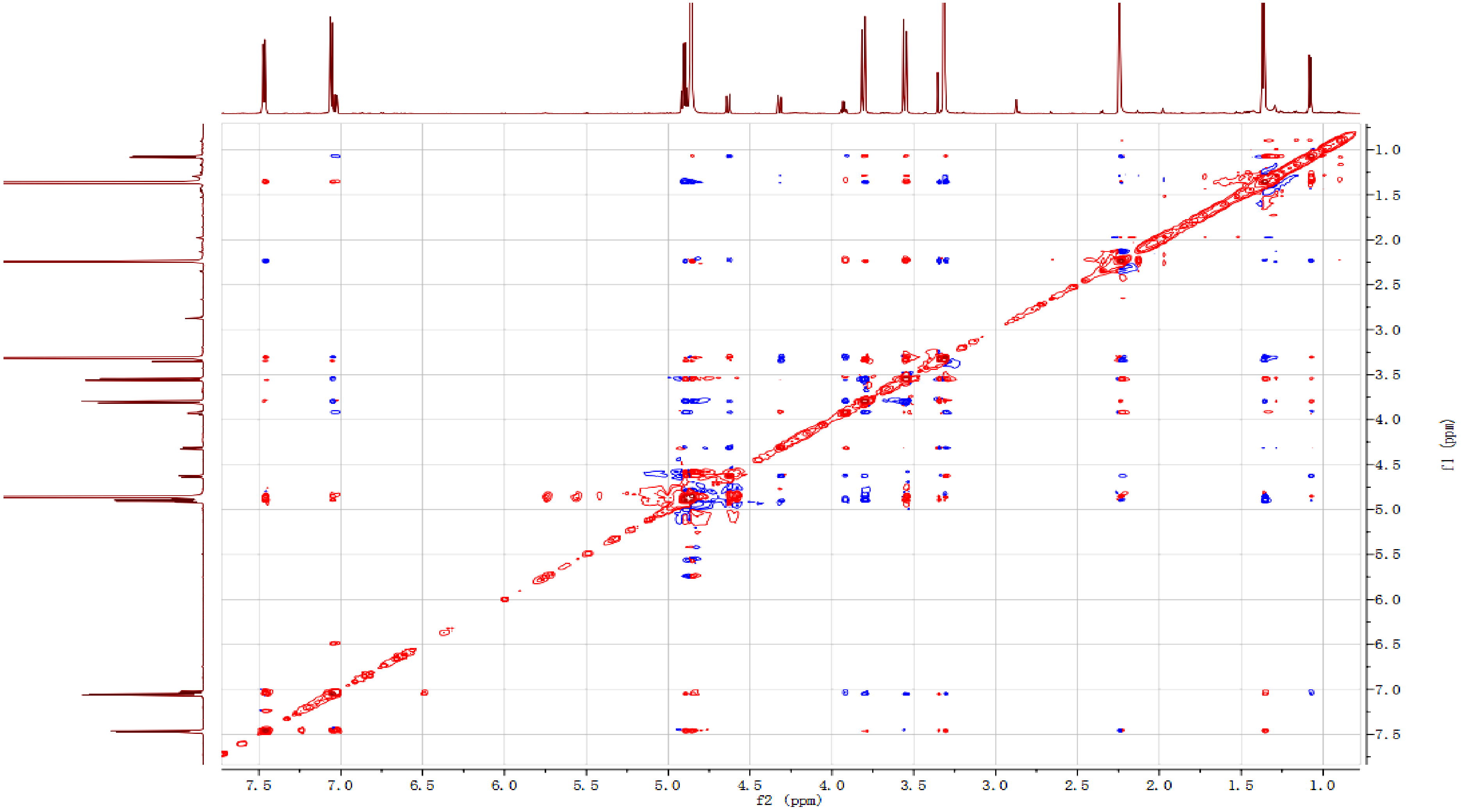

Figure S8. Nuclear overhauser effect spectroscopy (NOESY) spectrum of new compound **1**

Mass Spectrum Molecular Formula Report

Analysis Info

Analysis Name	D:\Data\20160322ceyang\neu-26_1-A,4_01_6801.d	Acquisition Date	3/22/2016 4:26:37 PM
Method	20131026_ceyang.m	Instrument / Ser#	Bruker Customer
Sample Name	neu-26	Operator	micrOTOF-Q 125
Comment			

Acquisition Parameter

Source Type	ESI	Ion Polarity	Negative	Set Nebulizer	1.2 Bar
Focus	Not active	Set Capillary	3800 V	Set Dry Heater	180 °C
Scan Begin	50 m/z	Set End Plate Offset	-500 V	Set Dry Gas	8.0 l/min
Scan End	1500 m/z	Set Collision Cell RF	400.0 Vpp	Set Divert Valve	Source

Generate Molecular Formula Parameter

Formula, min.		
Formula, max.		
Measured m/z	Tolerance	Charge
Check Valence	Minimum	Maximum
Nirogen Rule	Electron Configuration	
Filter H/C Ratio	Minimum	Maximum
Estimate Carbon		

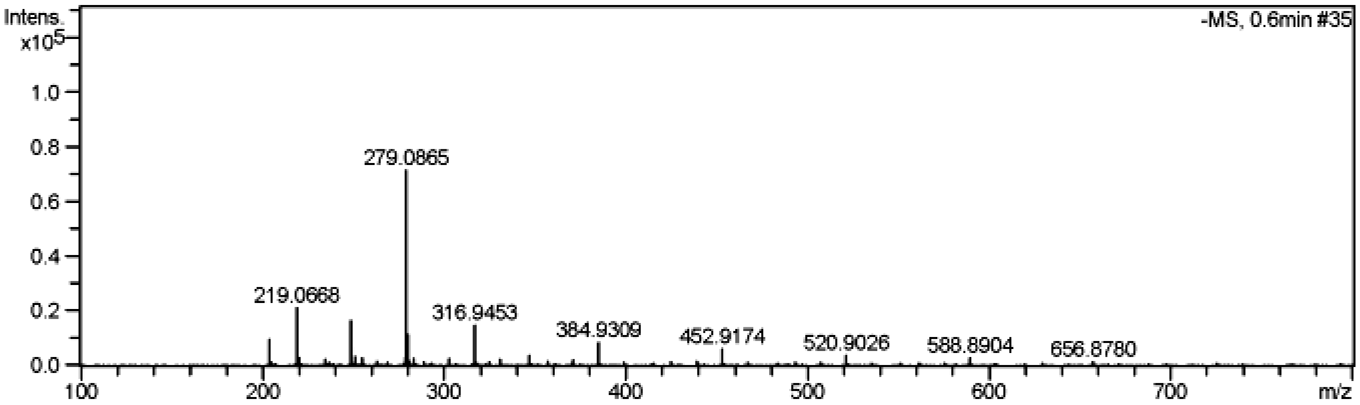

Figure S9. HRESI–MS spectrum of new compound **2**

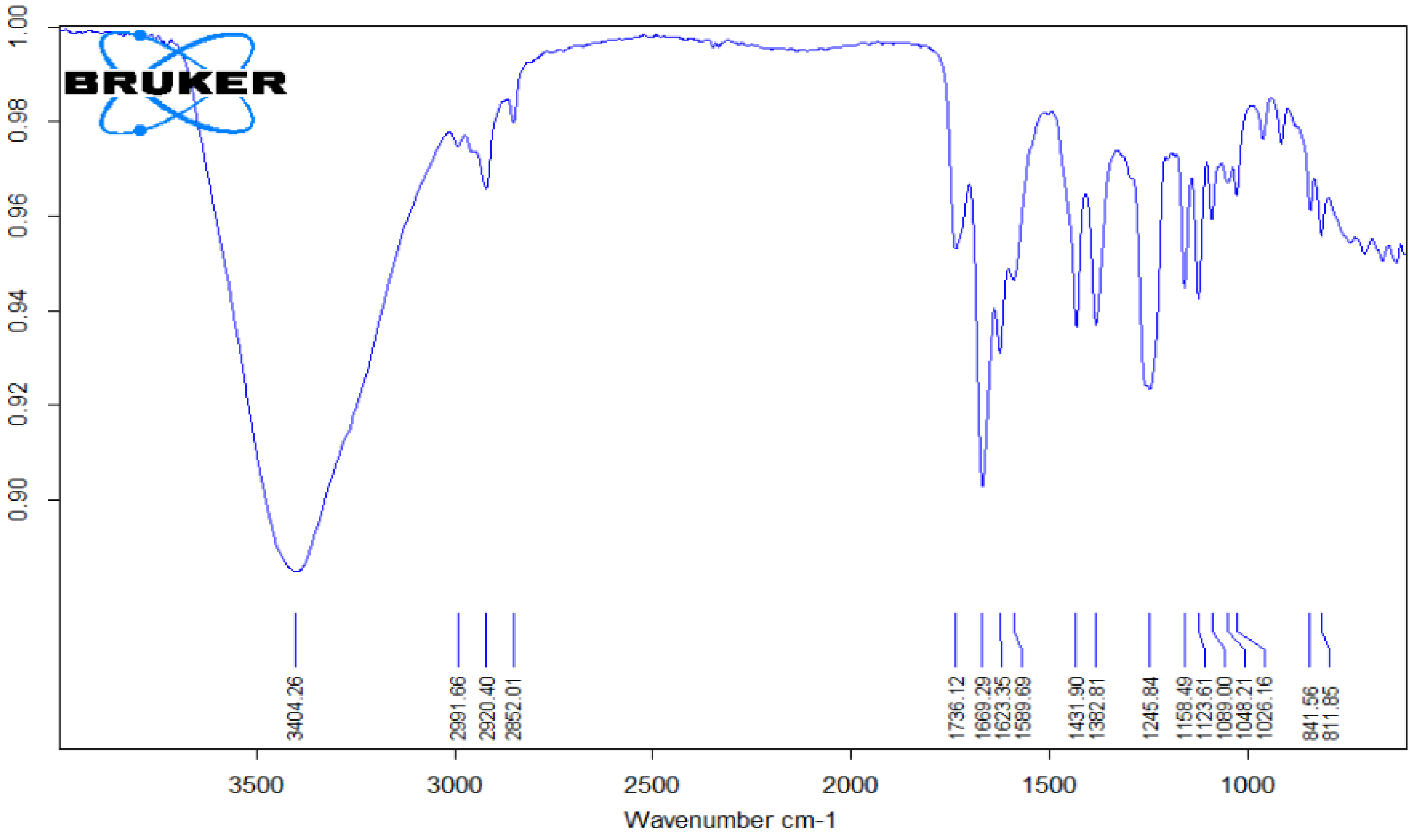

Figure S10. IR spectrum of the new compound **2**

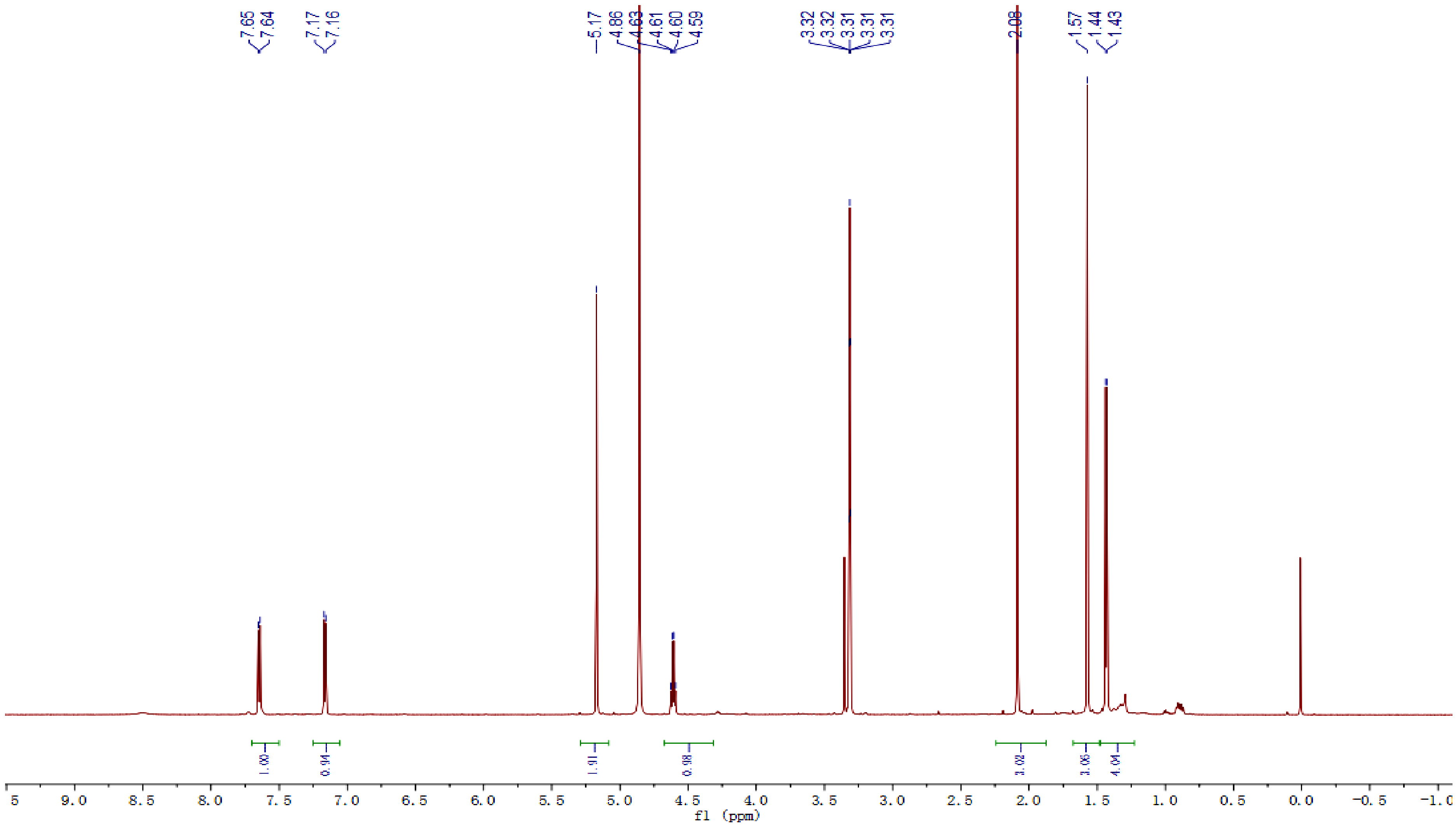

Figure S11. ^{1}H NMR (600 MHz, CD_3OD) spectrum of new compound **2**

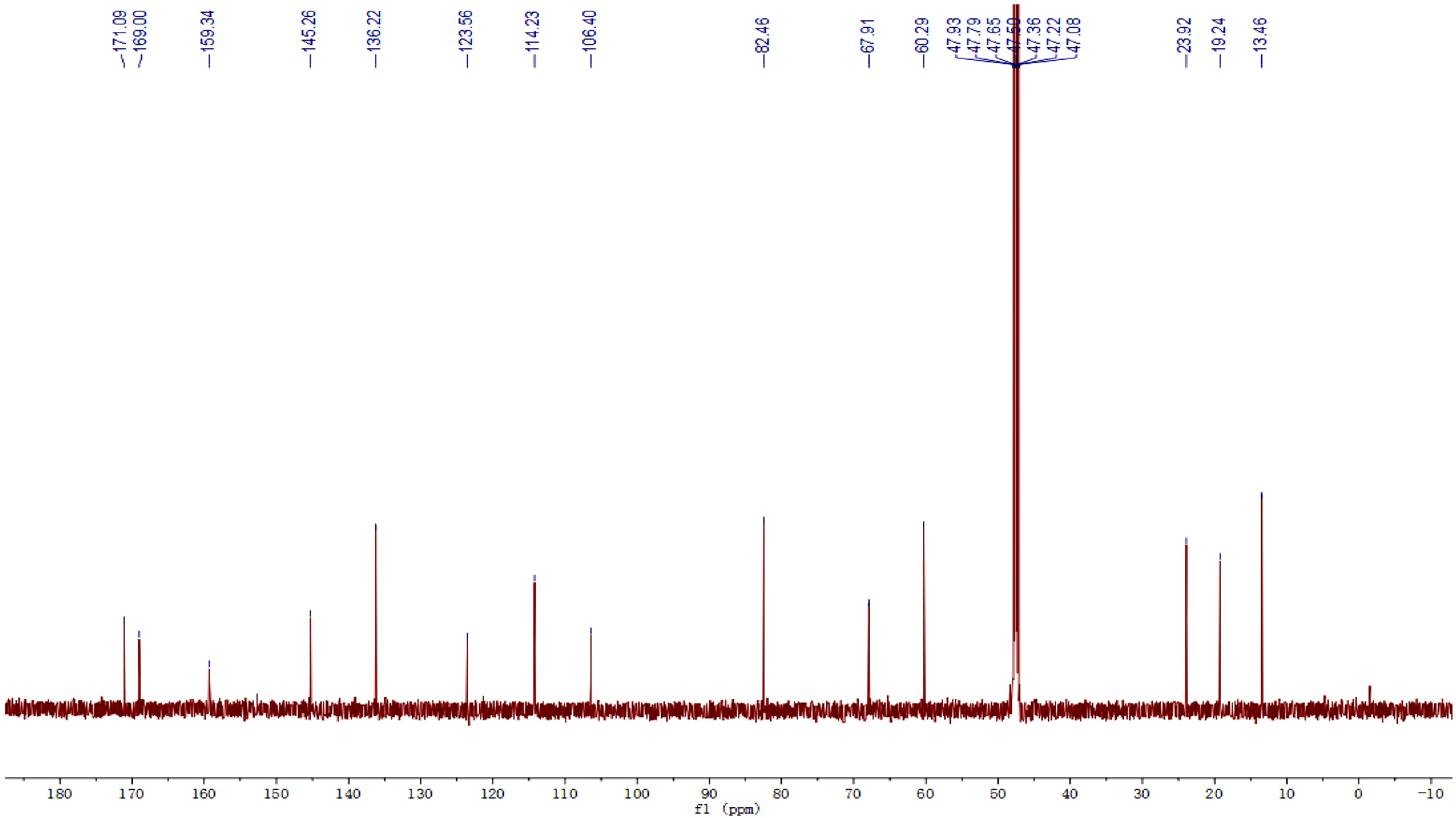

Figure S12. ^{13}C NMR (150 MHz, CD_3OD) spectrum of new compound **2**

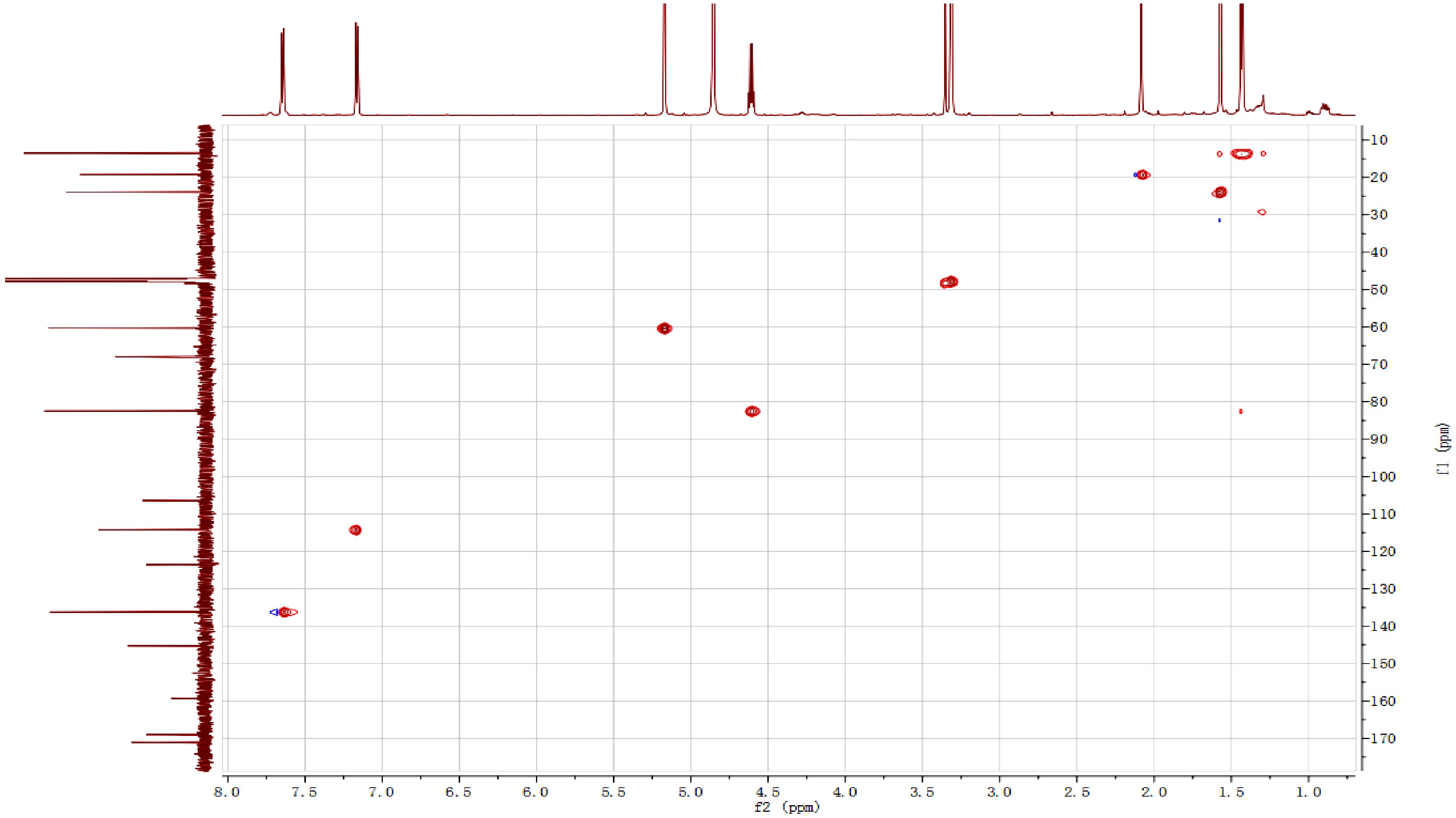

Figure S13. HSQC spectrum of new compound **2**

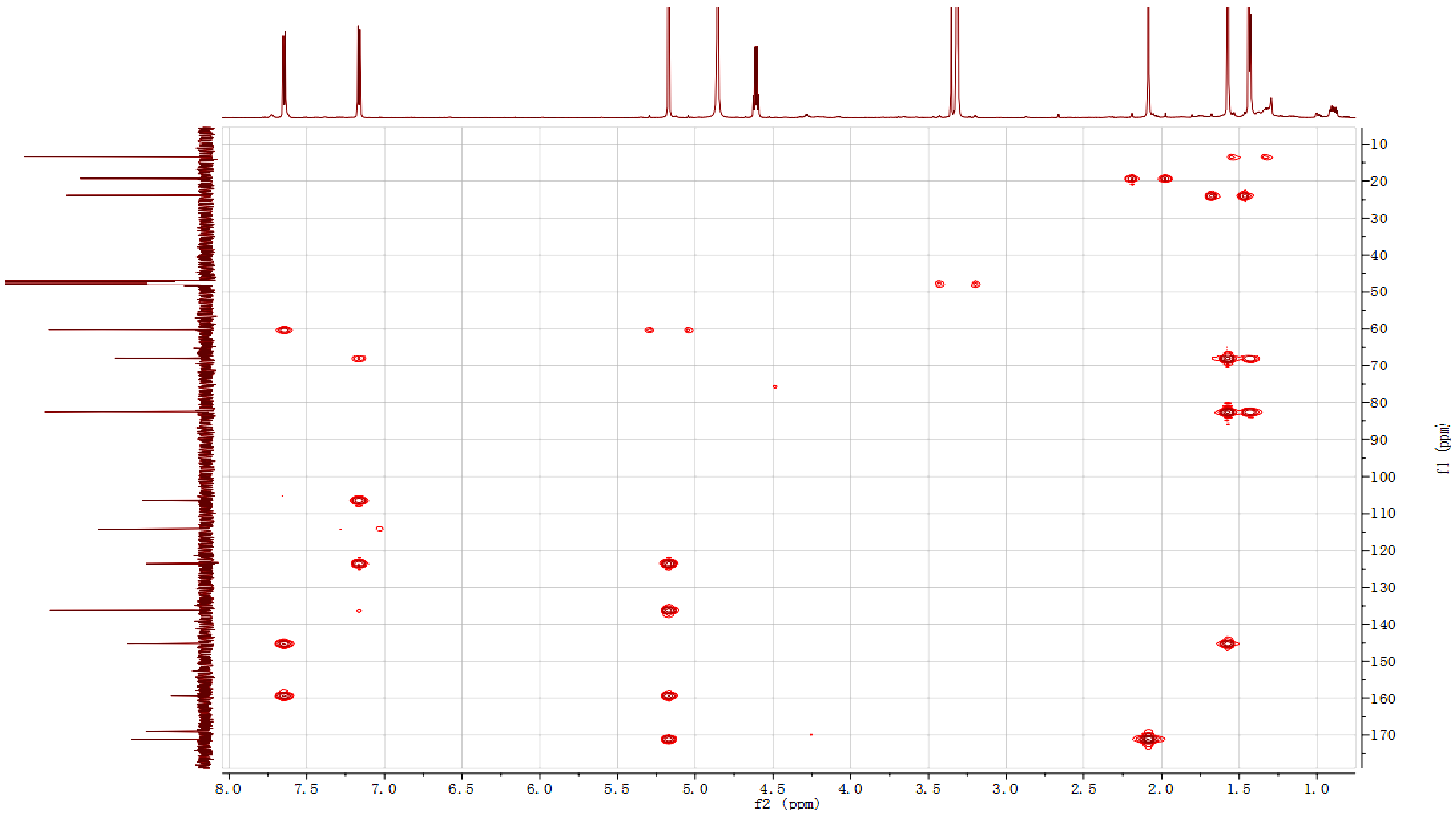

Figure S14. HMBC spectrum of new compound **2**

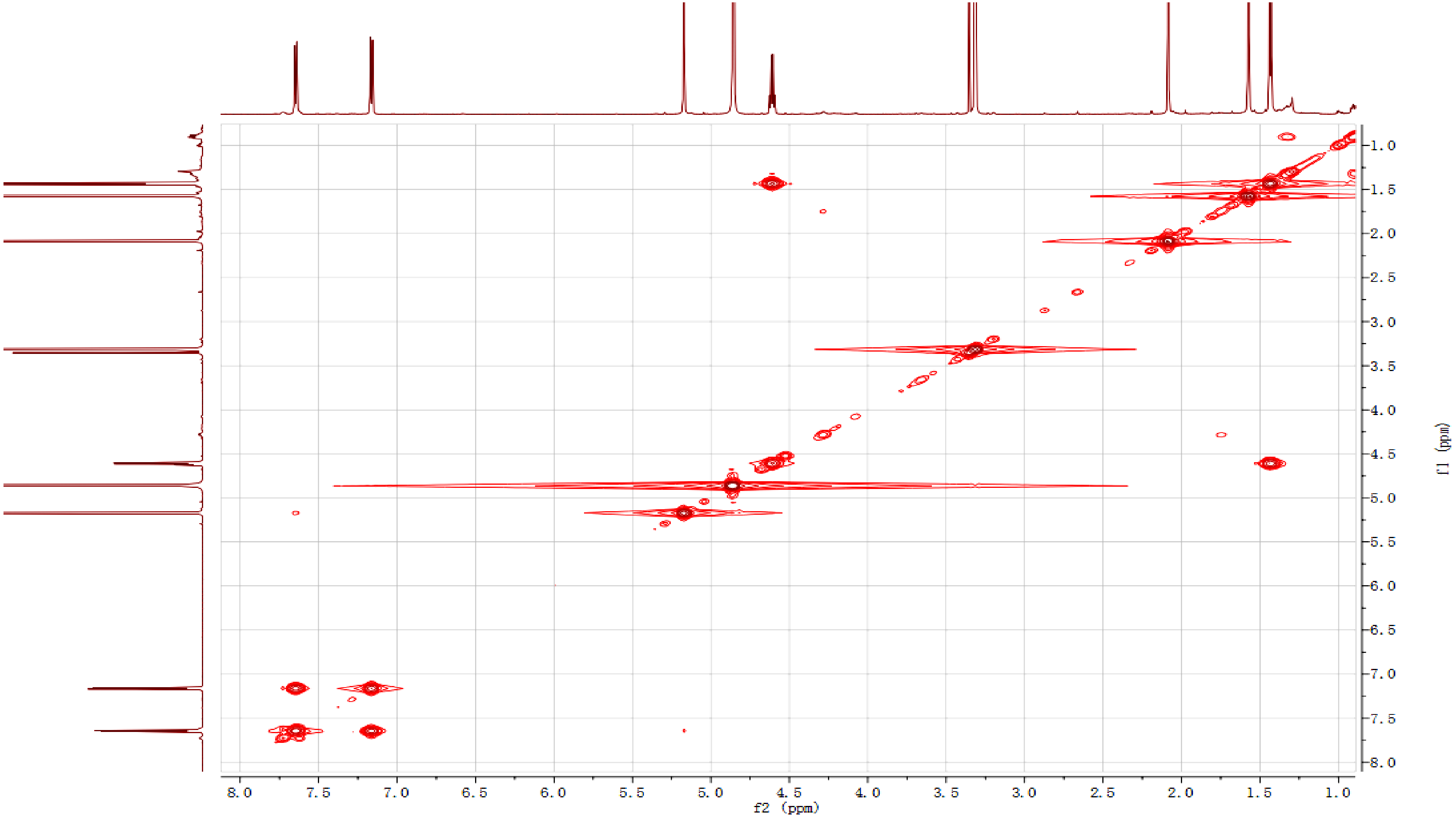

Figure S15. COSY spectrum of new compound **2**

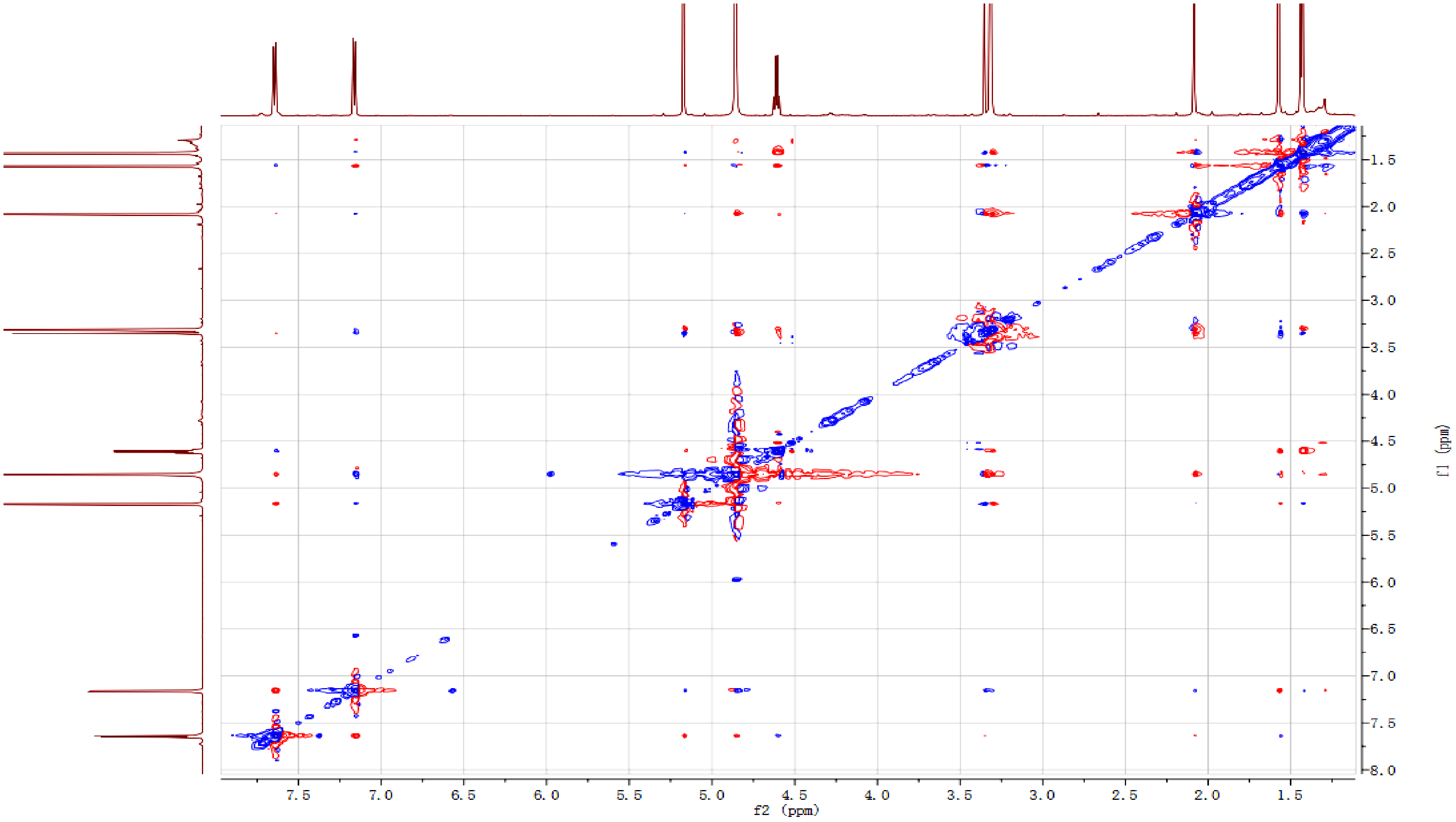

Figure S16. NOESY spectrum of new compound **2**

Mass Spectrum Molecular Formula Report

Analysis Info

Analysis Name	D:\Data\20151216CEYANG\new folder\NEU-14_1-B,5_01_6260.d	Acquisition Date	12/16/2015 3:12:51 PM
Method	20131026_ceyang.m	Instrument / Ser#	Bruker Customer
Sample Name	NEU-14	Operator	micrOTOF-Q 125
Comment			

Acquisition Parameter

Source Type	ESI	Ion Polarity	Positive	Set Nebulizer	1.2 Bar
Focus	Active	Set Capillary	4500 V	Set Dry Heater	180 °C
Scan Begin	50 m/z	Set End Plate Offset	-500 V	Set Dry Gas	8.0 l/min
Scan End	3000 m/z	Set Collision Cell RF	100.0 Vpp	Set Divert Valve	Source

Generate Molecular Formula Parameter

Formula, min.		
Formula, max.		
Measured m/z	Tolerance	Charge
Check Valence	Minimum	Maximum
Nirogen Rule	Electron Configuration	
Filter H/C Ratio	Minimum	Maximum
Estimate Carbon		

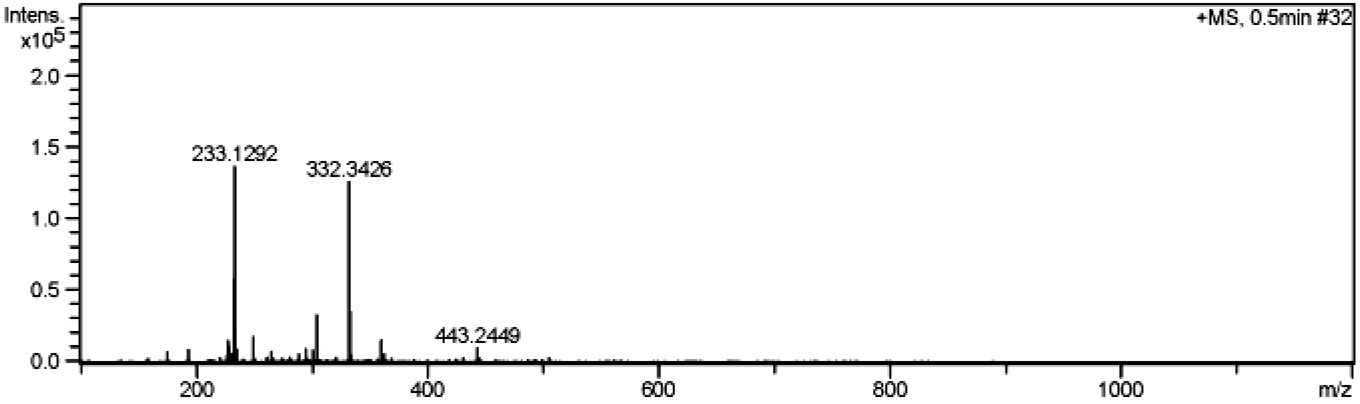

Figure S17. HRESI–MS spectrum of new compound **4**

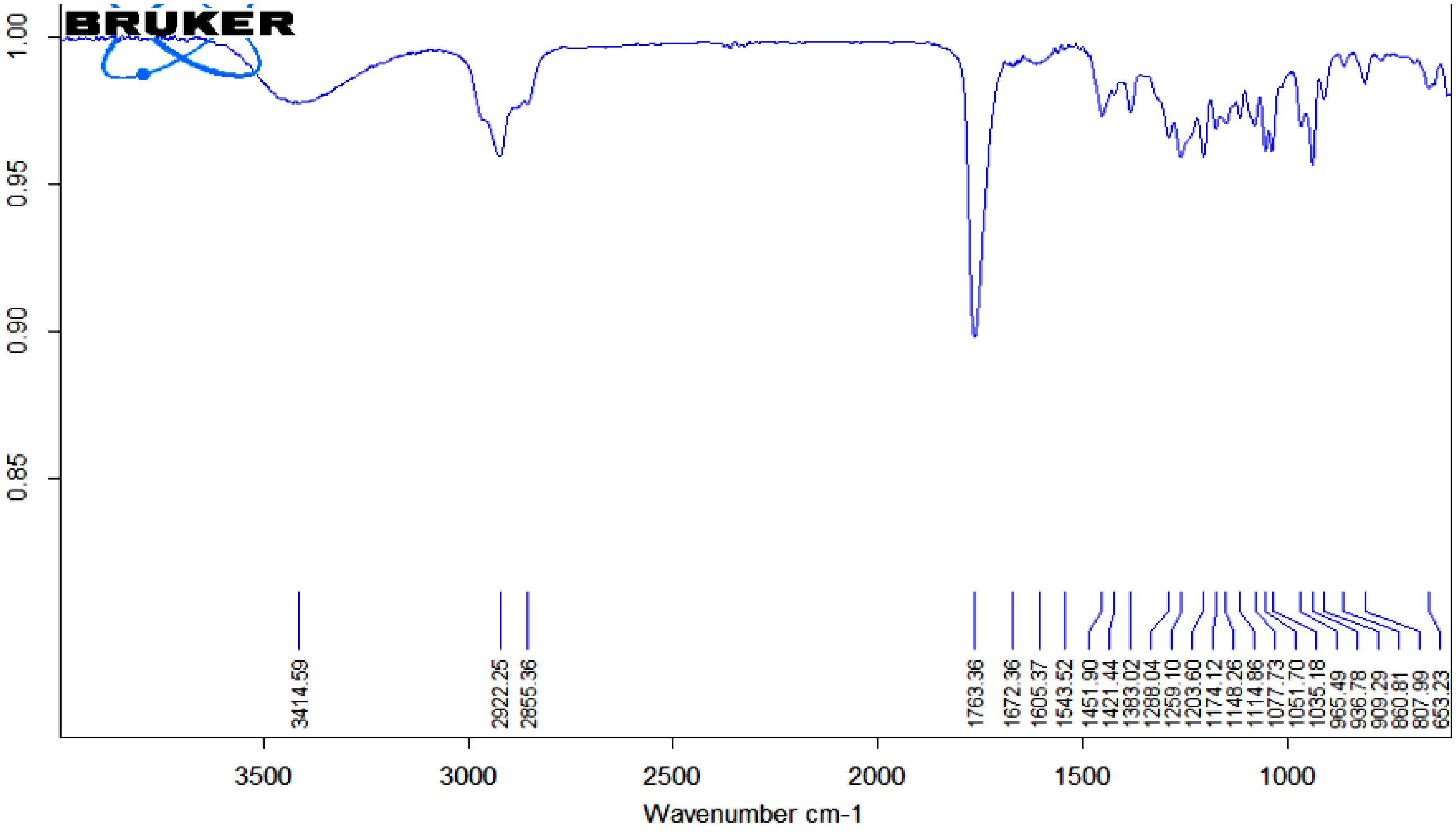

Figure S18. IR spectrum of new compound **4**

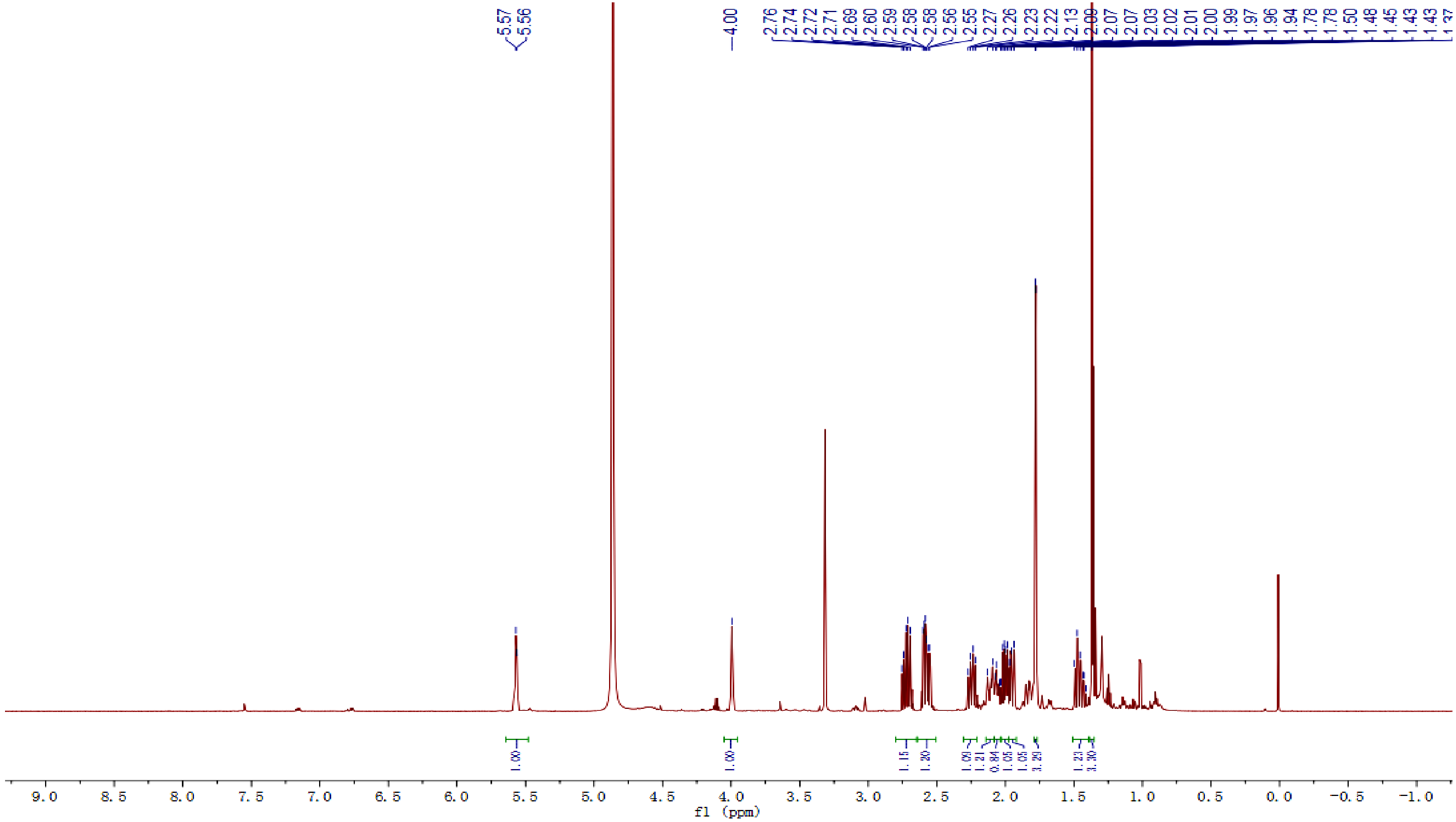

Figure S19. ^1H NMR (600 MHz, CD_3OD) spectrum of new compound **4**

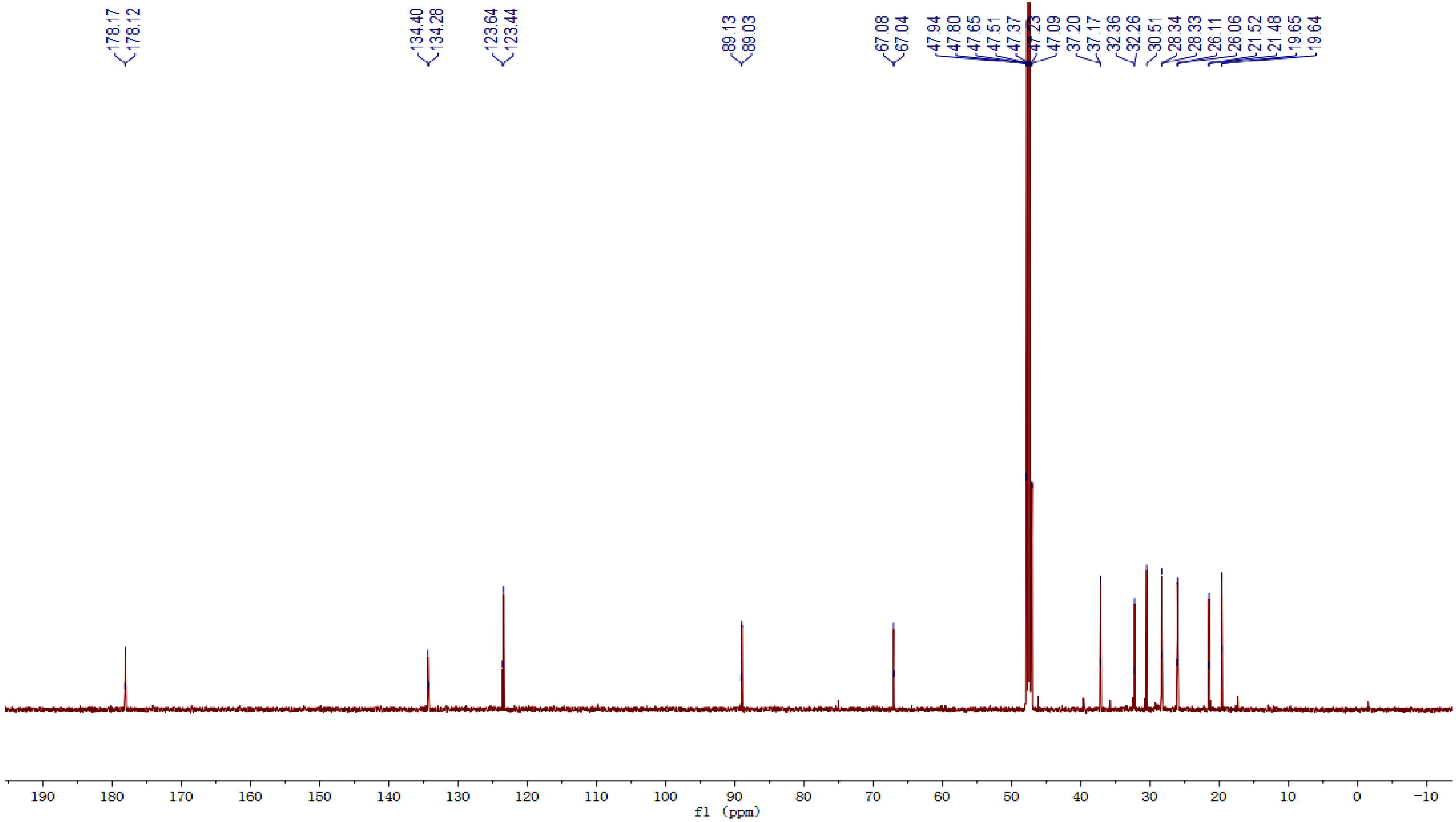

Fiure S20. ^{13}C NMR (150 MHz, CD_3OD) spectrum of new compound **4**

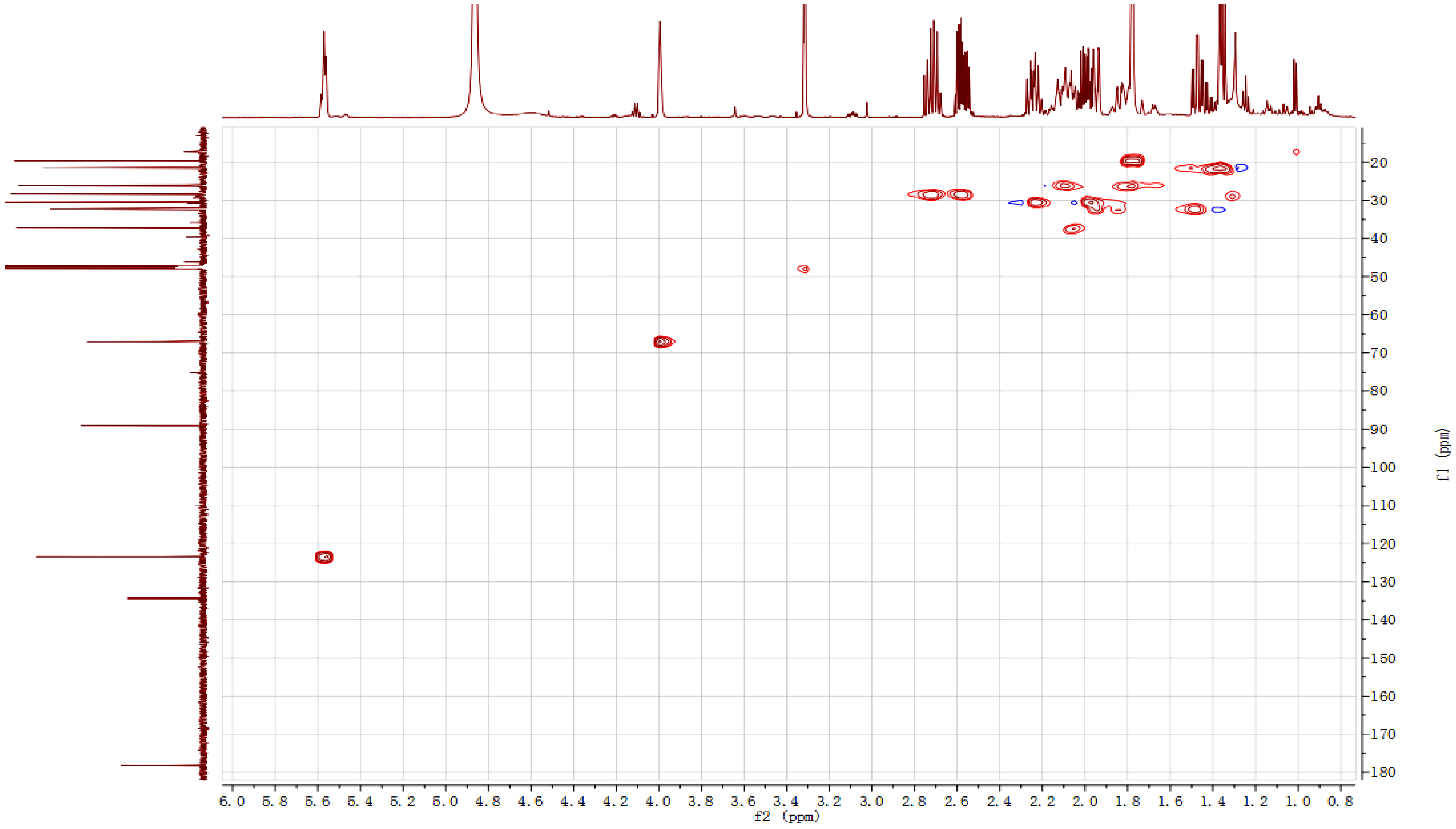

Figure S21. HSQC spectrum of new compound **4**

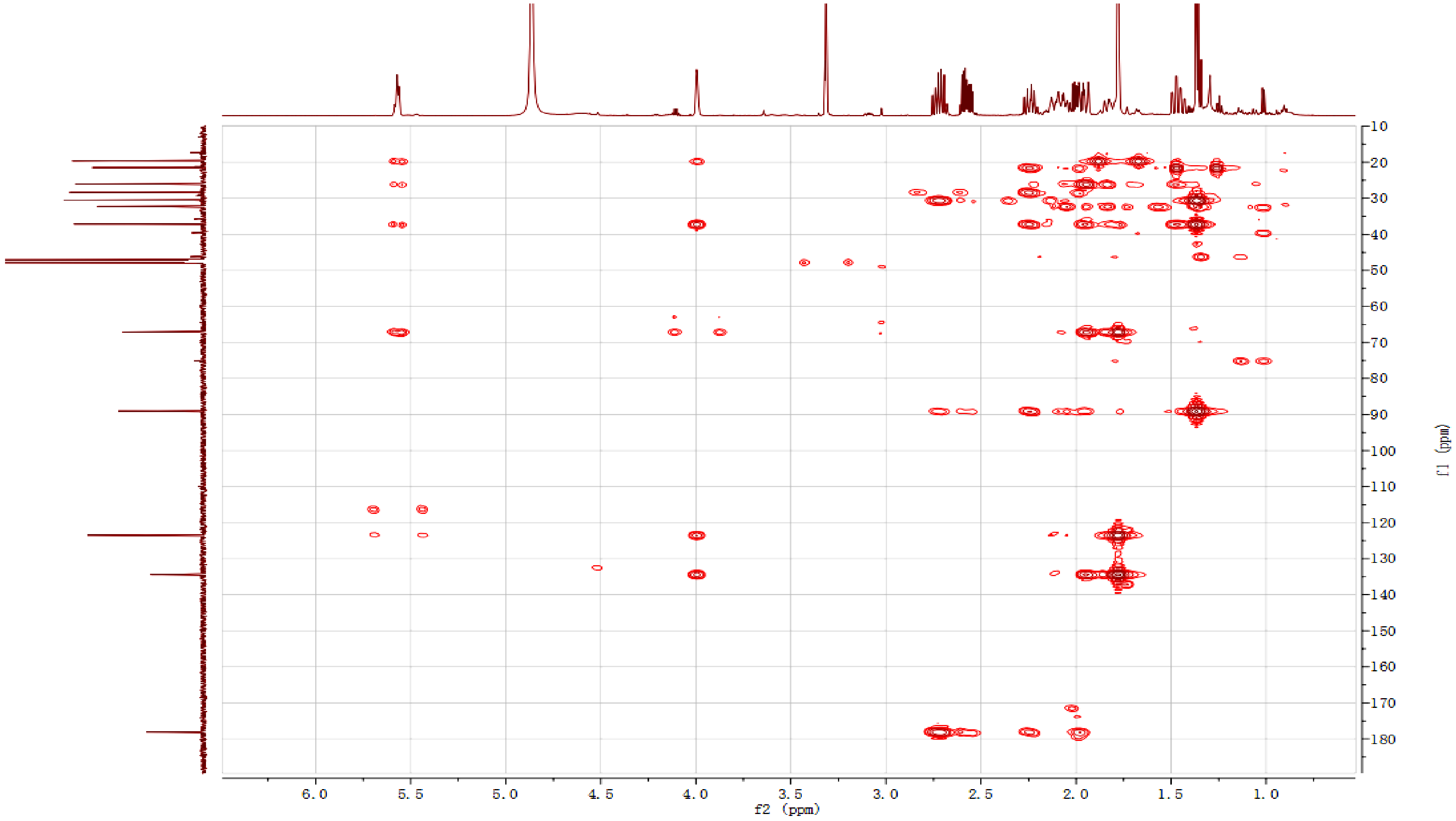

Figure S22. HMBC spectrum of new compound **4**

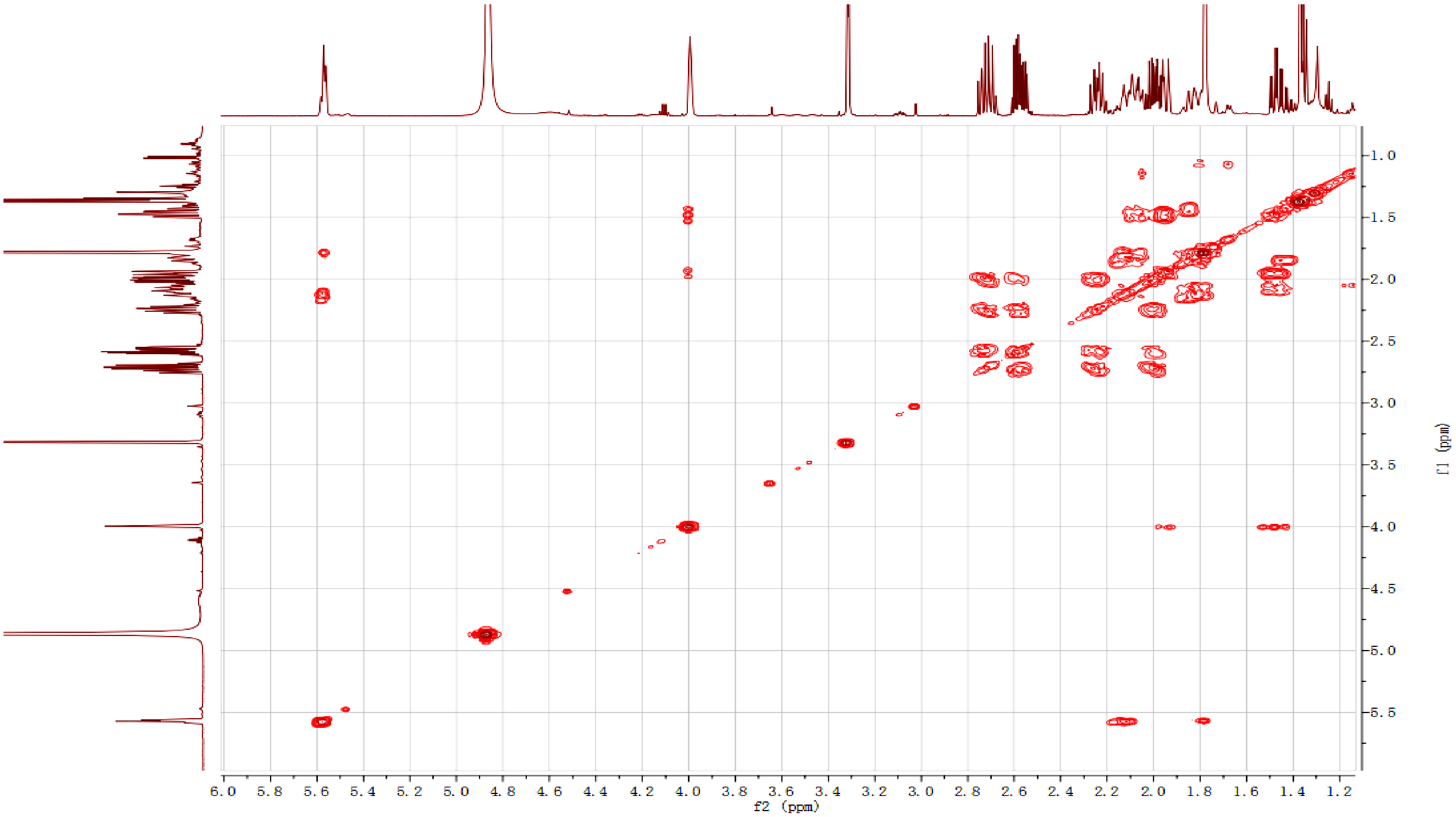

Figure S23. COSY spectrum of new compound **4**

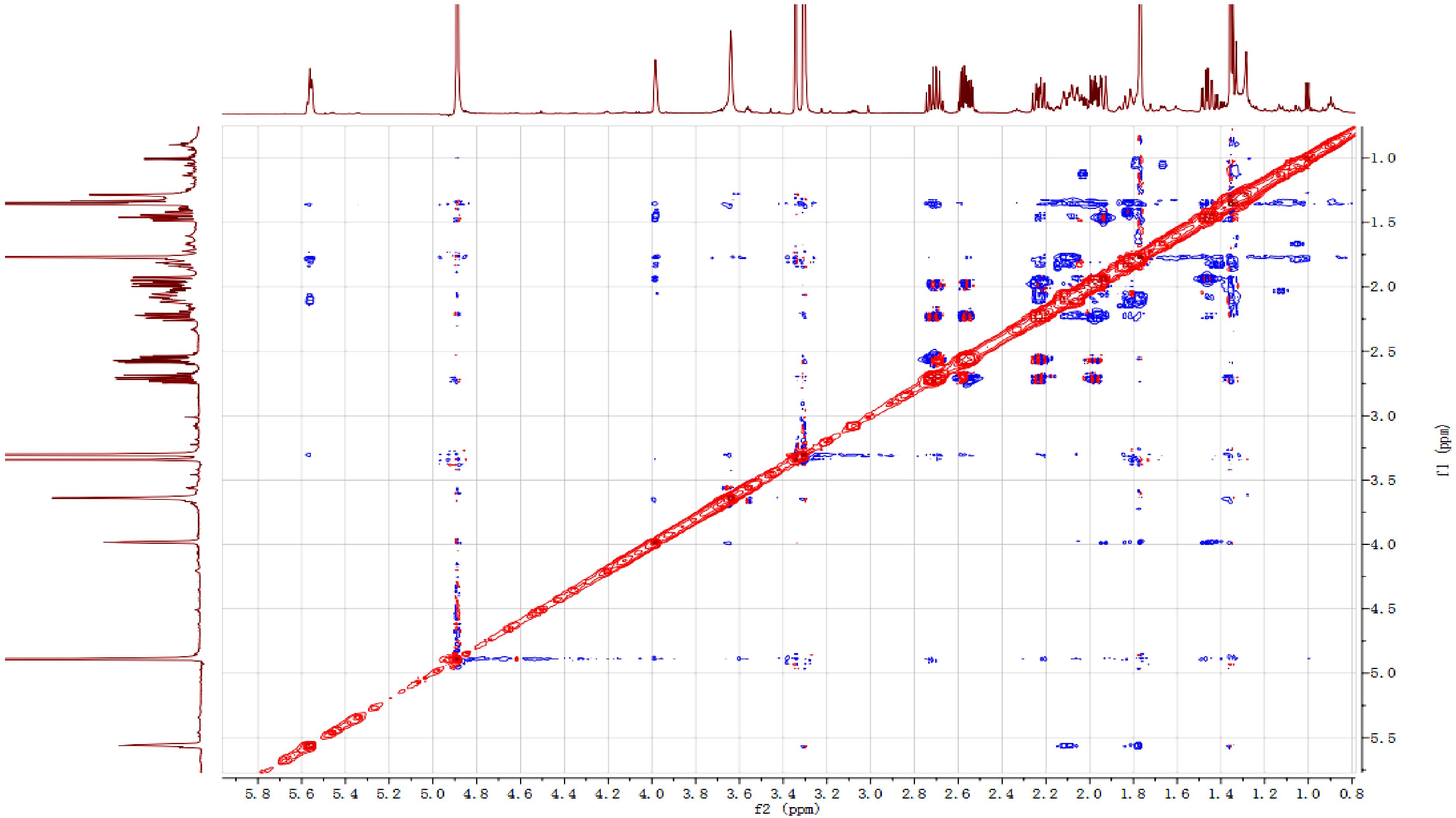

Figure S24. NOESY spectrum of new compound **4**

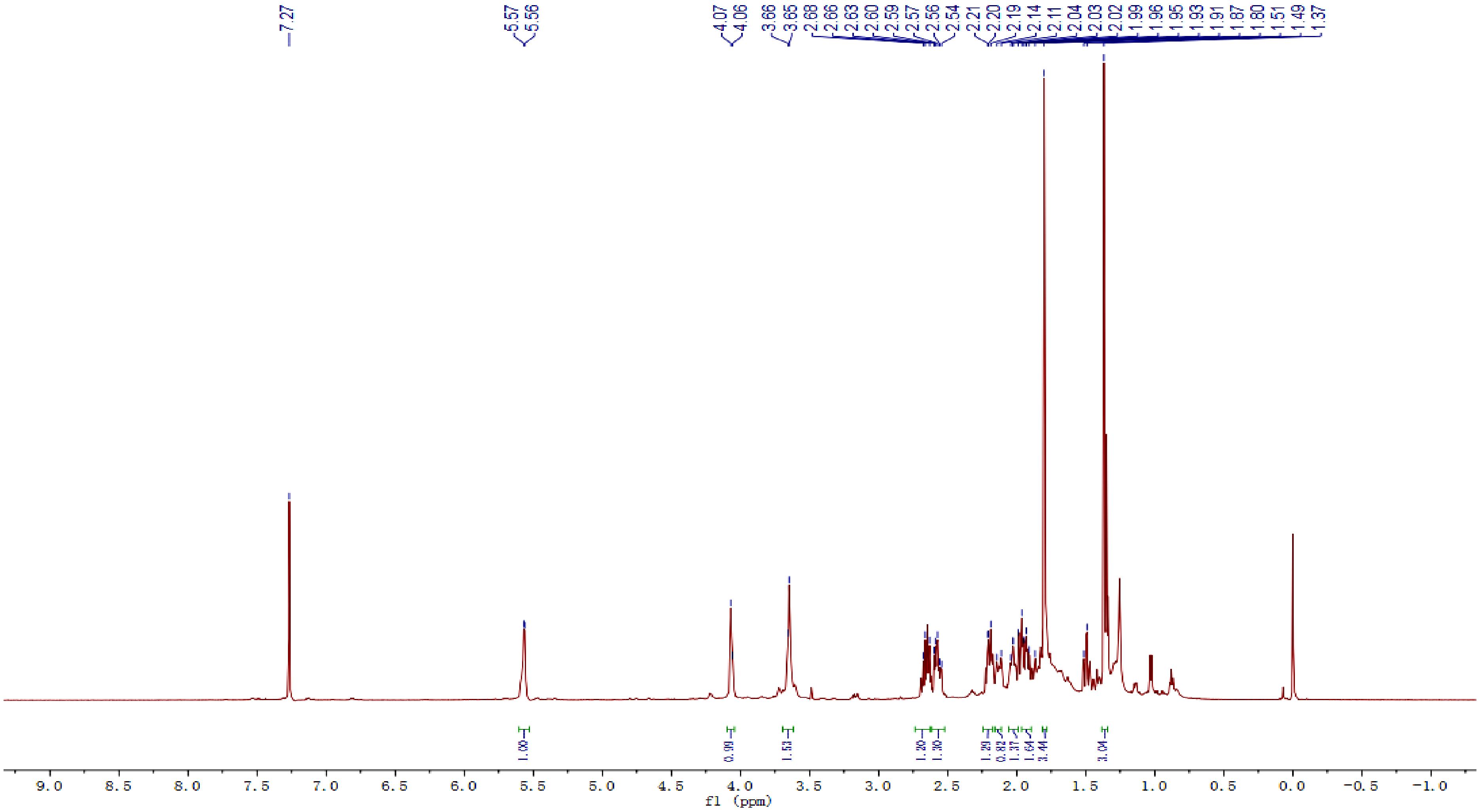

Figure S25. ^1H NMR (600 MHz, $CDCl_3$) spectrum of new compound **4**

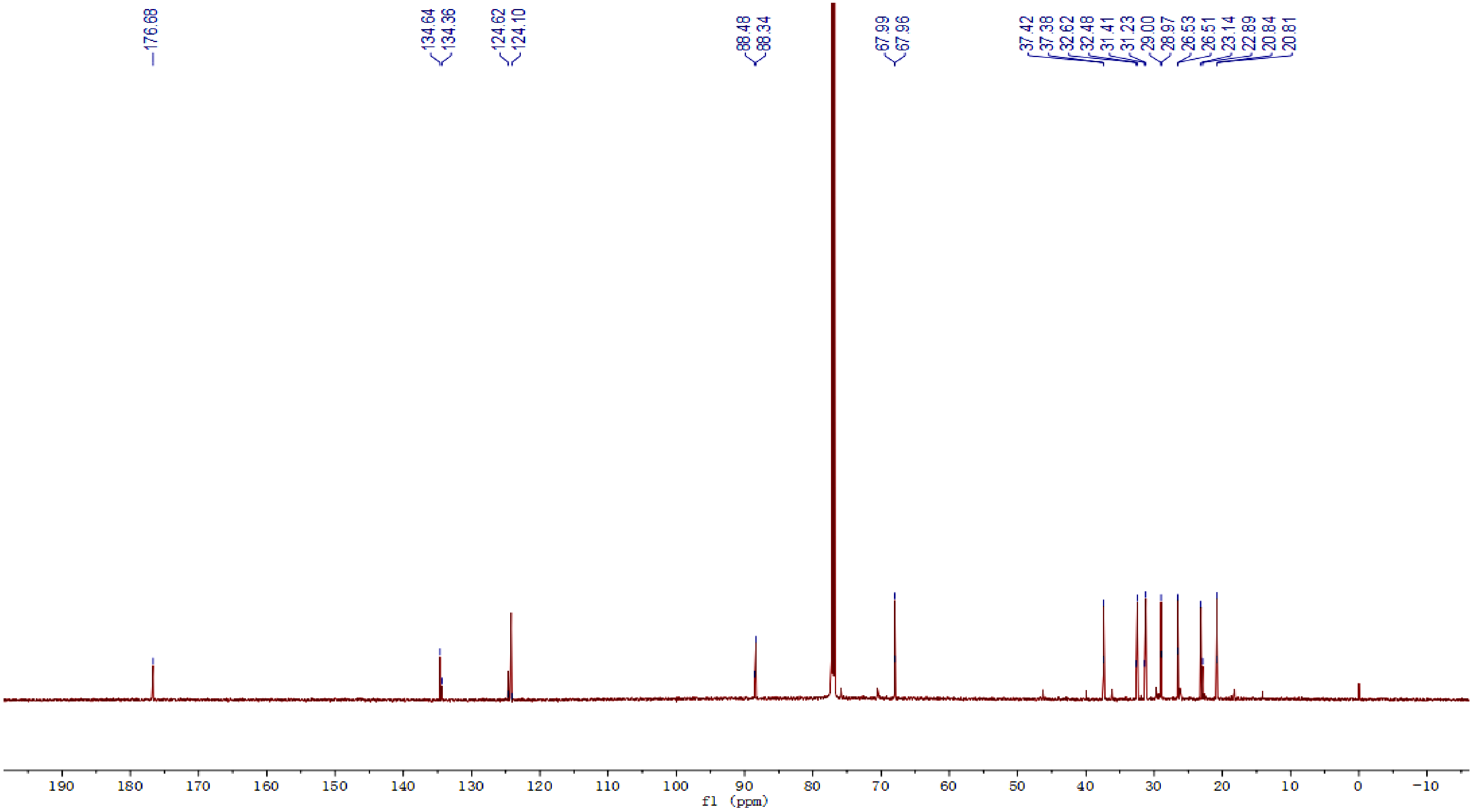

Figure S26. ^{13}C NMR (150 MHz, $CDCl_3$) spectrum of new compound **4**

Mass Spectrum Molecular Formula Report

Analysis Info

Analysis Name	D:\Data\20151216CEYANG\new folder\NEU-15_1-B,6_01_6261.d	Acquisition Date	12/16/2015 3:17:01 PM
Method	20131026_ceyang.m	Instrument / Ser#	Bruker Customer
Sample Name	NEU-15	Operator	micrOTOF-Q 125
Comment			

Acquisition Parameter

Source Type	ESI	Ion Polarity	Positive	Set Nebulizer	1.2 Bar
Focus	Active	Set Capillary	4500 V	Set Dry Heater	180 °C
Scan Begin	50 m/z	Set End Plate Offset	-500 V	Set Dry Gas	8.0 l/min
Scan End	3000 m/z	Set Collision Cell RF	100.0 Vpp	Set Divert Valve	Source

Generate Molecular Formula Parameter

Formula, min.		
Formula, max.		
Measured m/z	Tolerance	Charge
Check Valence	Minimum	Maximum
Nirogen Rule	Electron Configuration	
Filter H/C Ratio	Minimum	Maximum
Estimate Carbon		

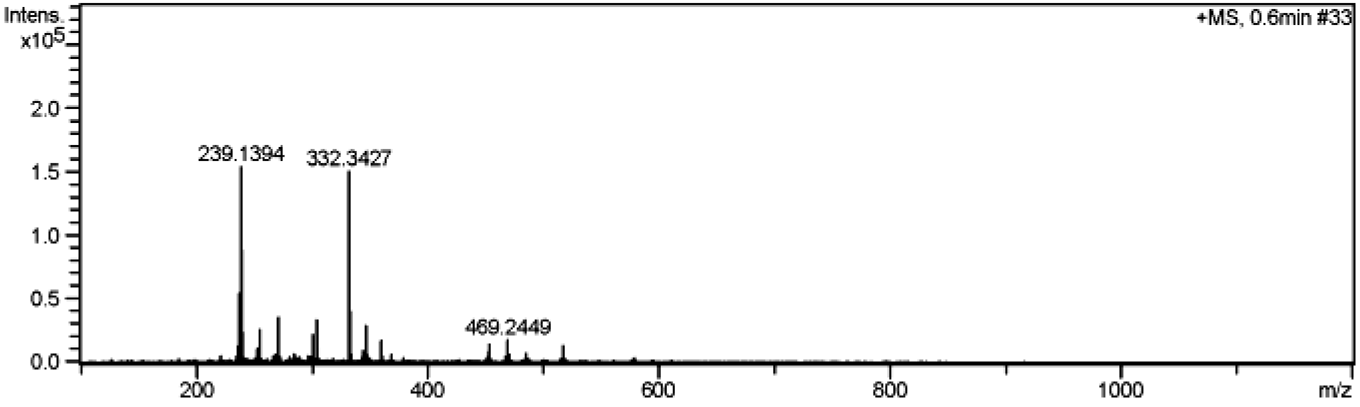

Figure S27. HRESI–MS spectrum of new compound **5**

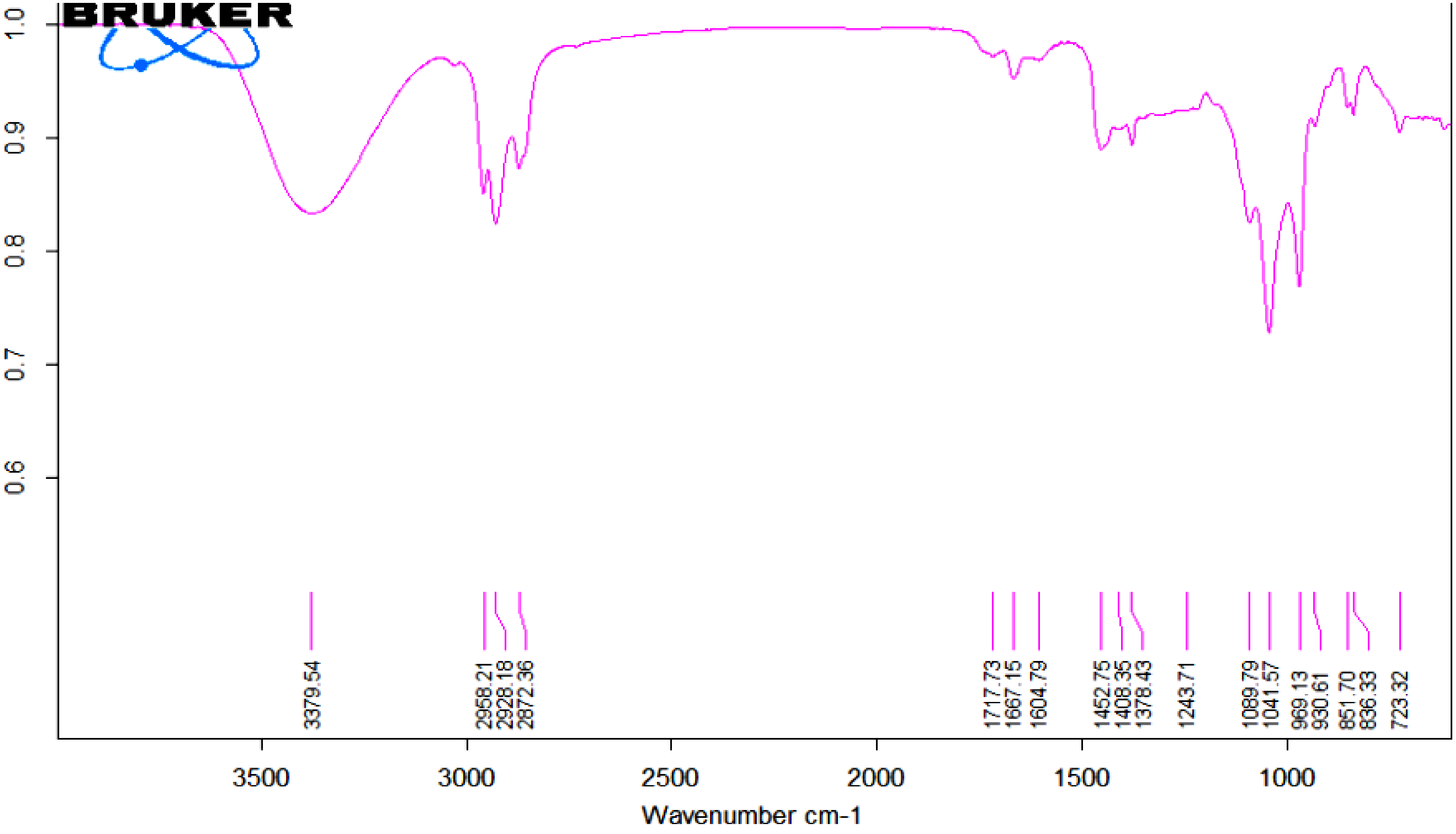

Figure S28. IR spectrum of new compound **5**

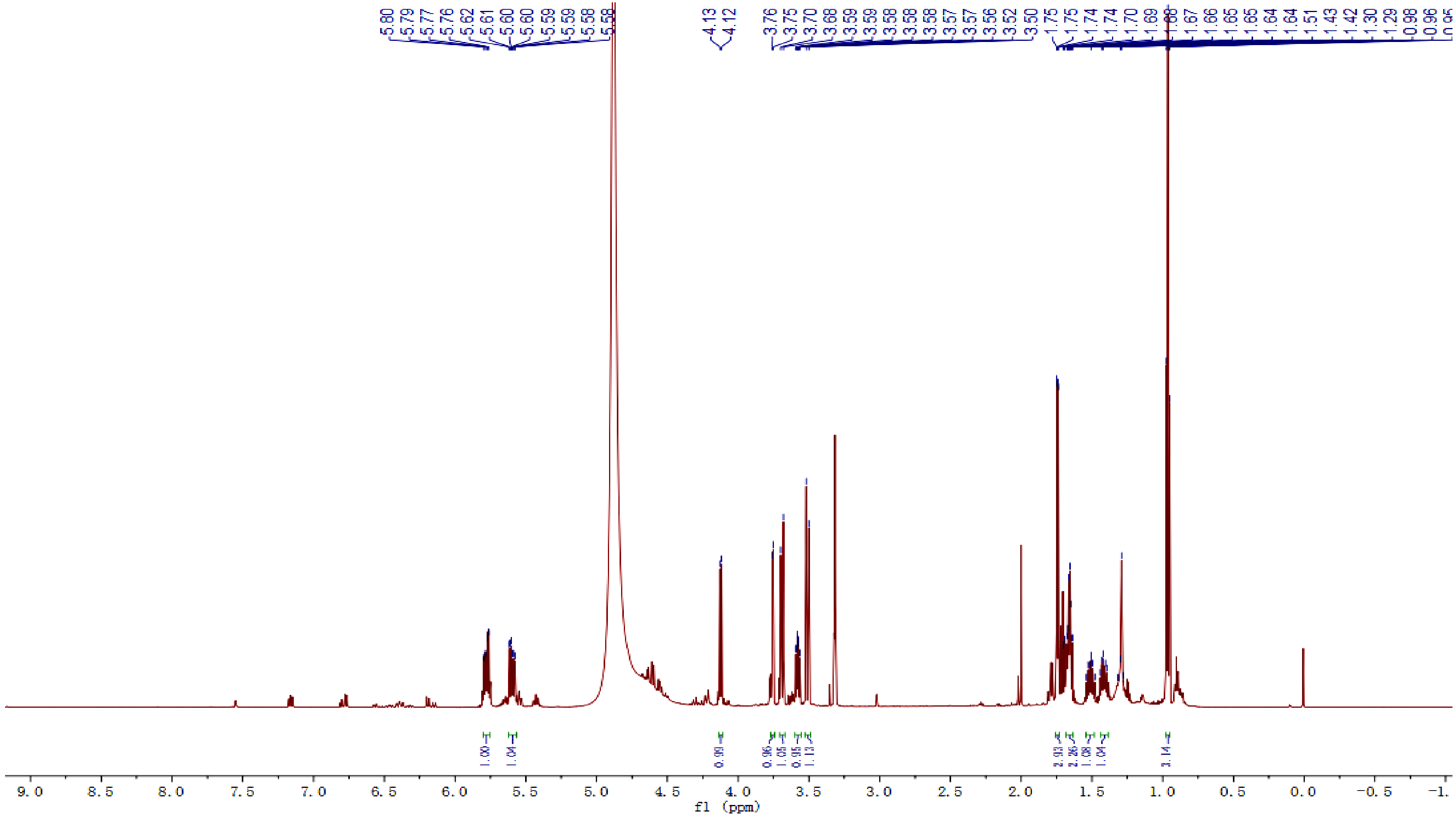

Figure S29. ^{1}H NMR (600 MHz, CD_3OD) spectrum of new compound **5**

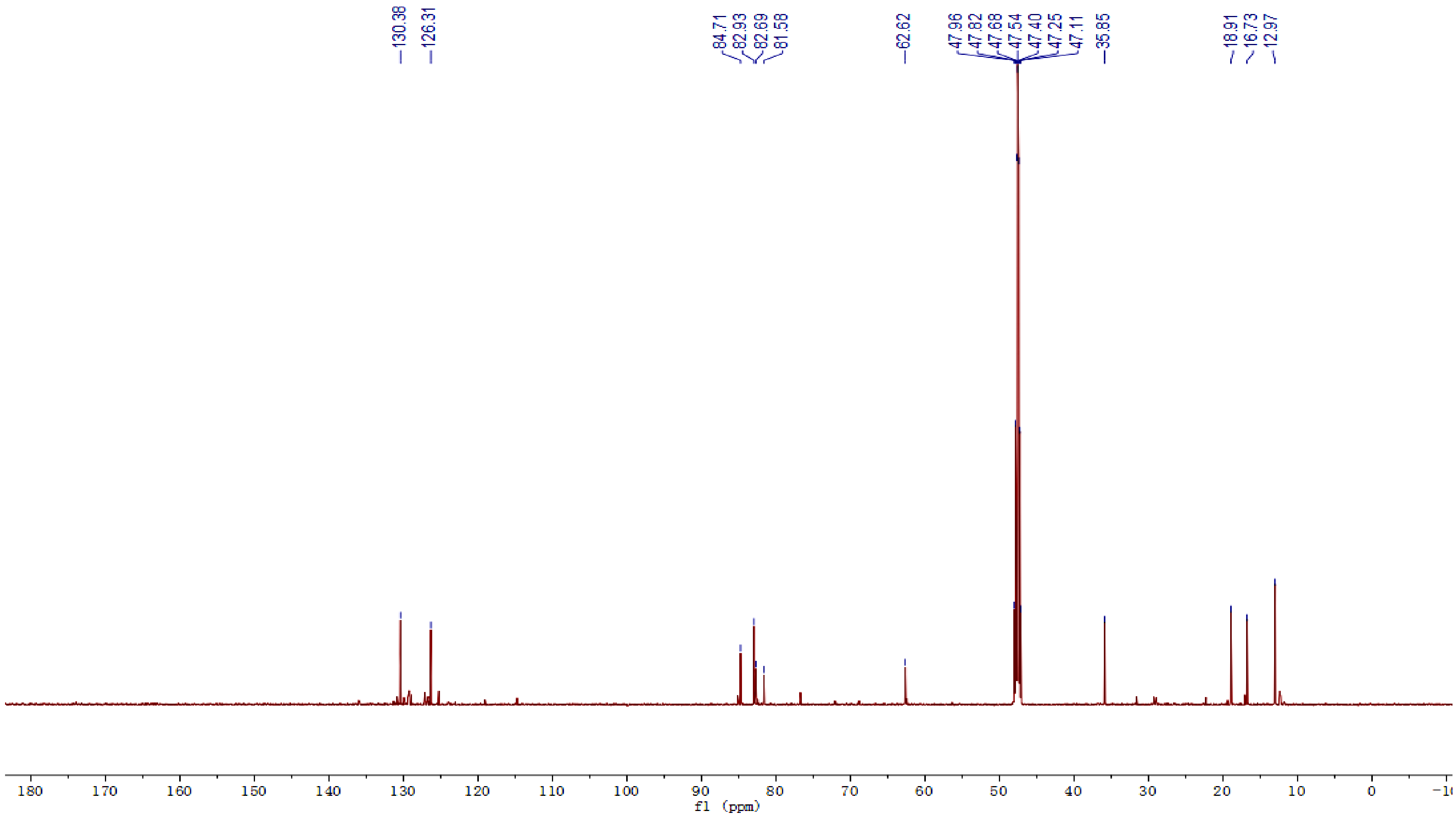

Figure S30. ^{13}C NMR (150 MHz, CD_3OD) spectrum of new compound **5**

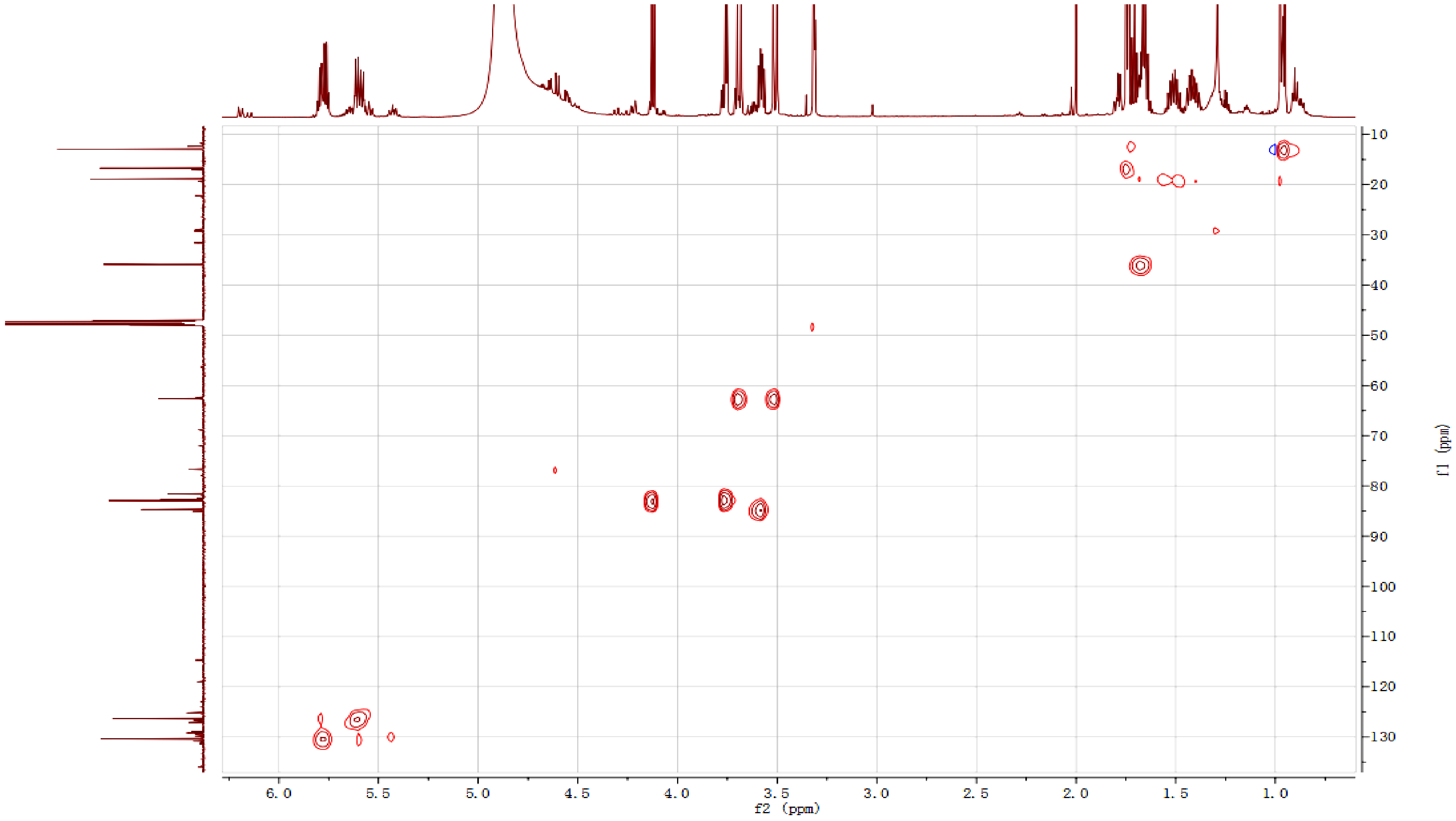

Figure S31. HSQC spectrum of new compound **5**

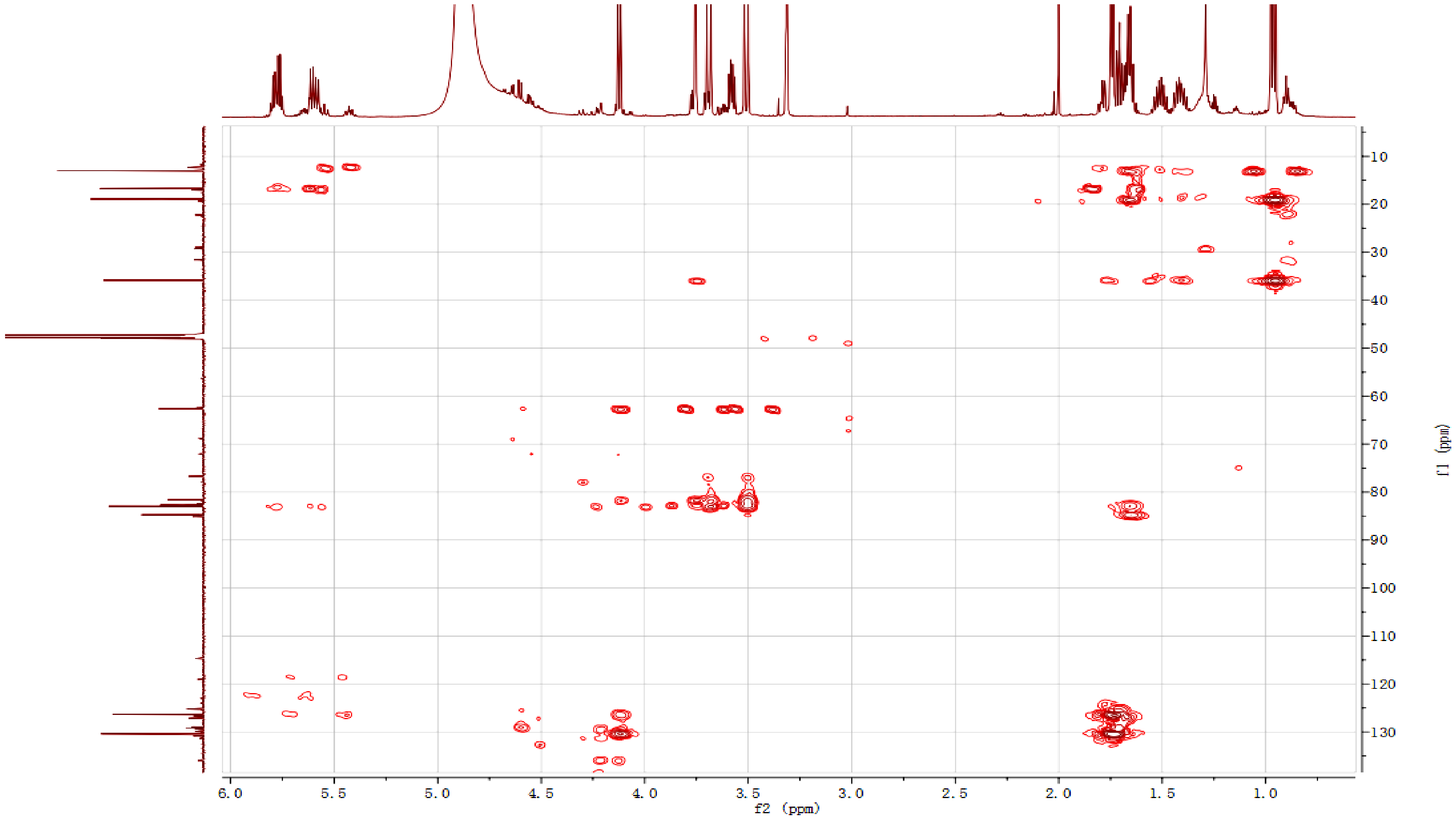

Figure S32. HMBC spectrum of new compound **5**

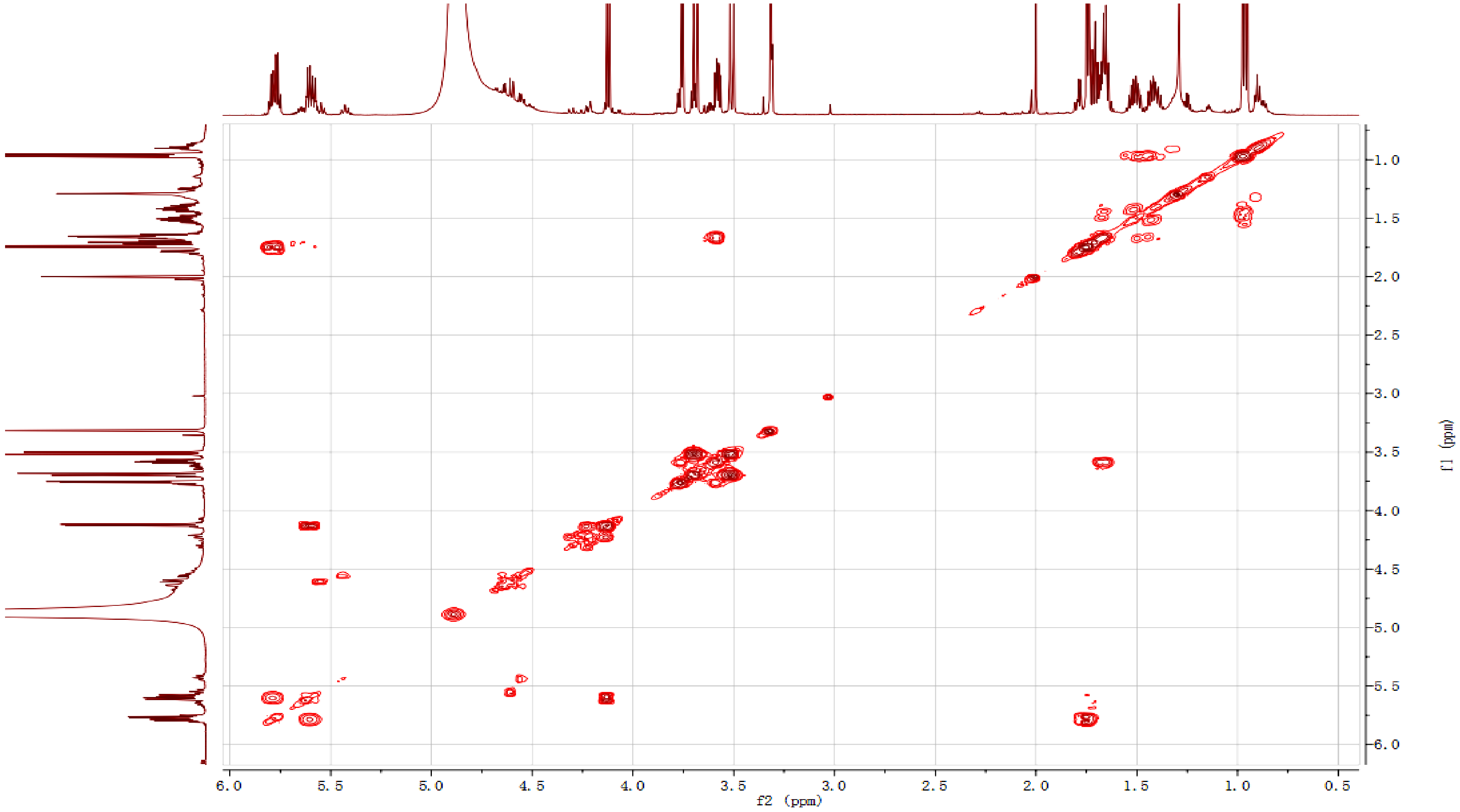

Figure S33. COSY spectrum of new compound **5**

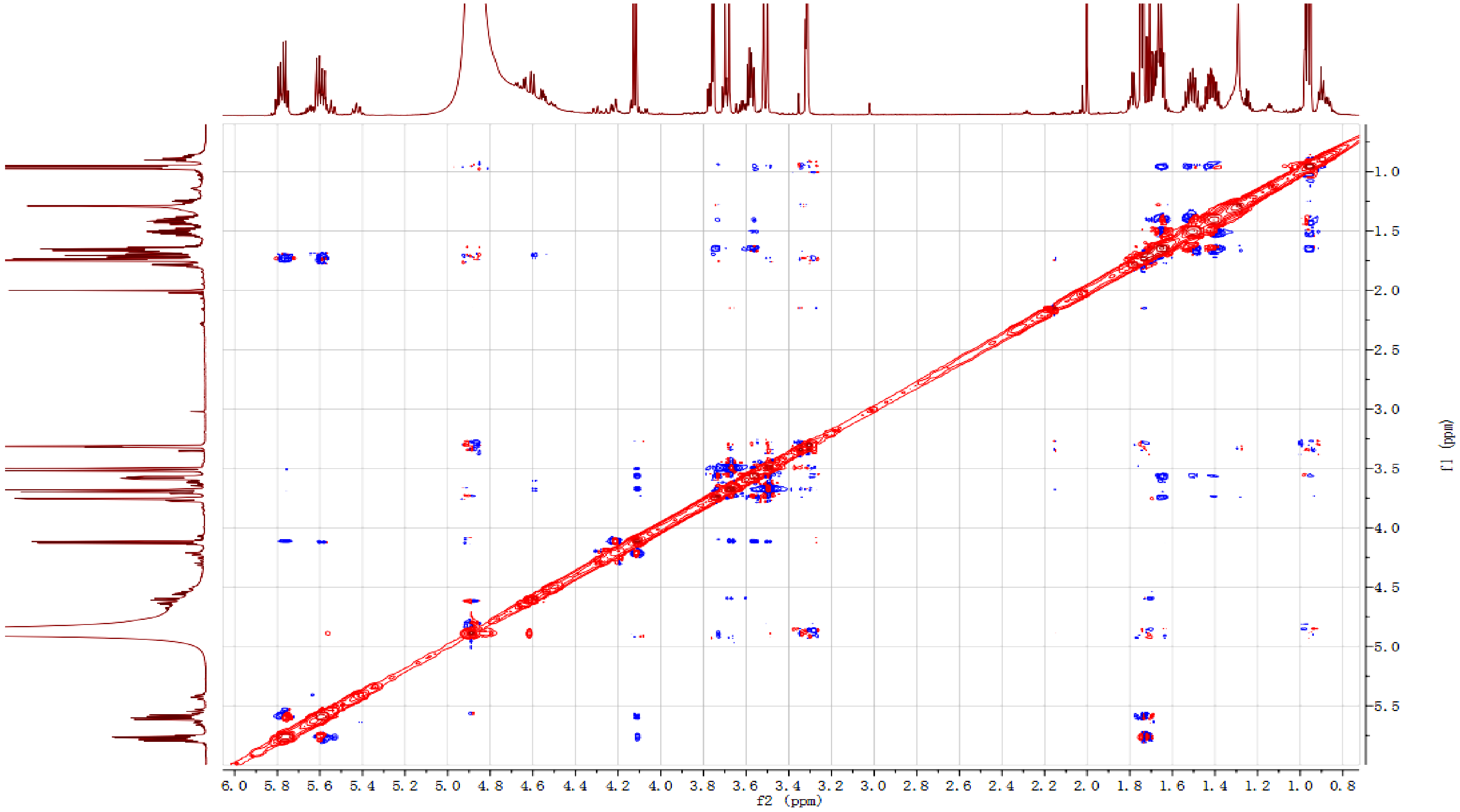

Figure S34. NOESY spectrum of new compound **5**
